# To vaccinate or not to vaccinate!? Predictors of willingness to receive Covid-19 vaccination in Europe, the U.S., and China

**DOI:** 10.1371/journal.pone.0260230

**Published:** 2021-12-01

**Authors:** Julia Brailovskaia, Silvia Schneider, Jürgen Margraf

**Affiliations:** 1 Mental Health Research and Treatment Center, Department of Clinical Psychology and Psychotherapy, Ruhr-Universität Bochum, Bochum, Germany; 2 Mental Health Research and Treatment Center, Department of Clinical Child and Adolescent Psychology, Ruhr-Universität Bochum, Bochum, Germany; National Cheng Kung University College of Medicine, TAIWAN

## Abstract

Researcher teams around the globe including the “Project Lightspeed” are intensively working on vaccines to fight the Covid-19 pandemic. However, the availability of effective vaccines does not guarantee the vaccination willingness among the population. In spring 2021, we investigated the vaccination willingness and its potential predictors in representative online samples in nine countries (China, France, Germany, Poland, Russia, Spain, Sweden, U.K., U.S.). Of the 9,264 participants, 79.9% revealed Covid-19 vaccination willingness. The highest willingness was in the U.K., followed by Spain and China, the lowest in Russia. In most countries, the perception of governmental Covid-19 measures as useful and the use of television reports as Covid-19 information source positively predicted the willingness. Further factors such as demographic variables, mental and physical health status, evaluation of governmental communication, social media use, and general adherence to Covid-19 measures showed a country-specific predictive pattern. Recommendations how to increase the vaccination willingness are provided.

## Introduction

In mid-January 2020, the German biotechnology company Biopharmaceutical New Technologies (BioNTech) started the “Project Lightspeed” in cooperation with two other companies Pfizer and Fosun [1]. The project’s main aim was a rapid development of an effective and well-tolerated vaccine against the coronavirus disease (Covid-19; severe acute respiratory syndrome coronavirus 2, SARS-CoV-2) following highly scientific and ethical standards. In November 2020, safety data were available for at least two months after the second dose of the vaccine [1–3]. Also, other researcher teams around the globe work intensively on the rapid development of vaccines against Covid-19. In September 2021, there were over 140 candidate vaccines undergoing clinical trials and about 20 approved vaccines worldwide [4, 5].

The enormously rapid development of safe and highly effective Covid-19 vaccines is one of the most impressive achievements of biomedical research in recent times. The success of the vaccination campaigns depends on the availability of the vaccines and their allocation that is positively associated with the income of a specific country [6]. But above all it depends on the willingness of the population to be vaccinated. The willingness can contribute to the achieving of a herd immunity without the infection of a large proportion of the population [7]. A herd immunity threshold of about 71–74% is required to overcome the pandemic outbreak and to return to “normal” life [see 8].

A large cross-national study reported an overall vaccination willingness of about 71.5% in the end of June 2020 in 19 different countries [9]. With 88.62% China was the country with the highest willingness; Russia was the country with the lowest willingness with 54.85%. In the other countries, the willingness was within this range. For example, it was 75.42% in the U.S., 74.33% in Spain, 71.48% in the U.K., 68.74% in Germany, 65.23% in Sweden, 58.89% in France, and 56.31% in Poland [9]. Further country-specific research revealed similar vaccination willingness rates in these countries. In the beginning of the summer 2020, the willingness ranged between 82–83% in China [10, 11]. In April 2020, it was about 75% in the U.S. [12], about 70% in Germany and about 62% in France [8]. However, there were also studies that provided varying ranges within some countries. In the U.K., the willingness ranged between 79% in April 2020 [8] and 64% in July 2020 [13]. In Germany, a willingness of 83.6% was assessed in December 2020 [14] and of 78.3% in January 2021 [15]. In the U.S., a vaccination willingness of 71% was measured in April 2020, that decreased down to 53.6% in October 2020 [16]. In Russia, a willingness of 41.7% was assessed in autumn 2020 [17]. In Spain, a willingness of 48.3% was reported in winter 2020 [18].

Thus, the vaccination willingness seems to differ between the countries, and within the same country depending on the period of data collection. This emphasizes the need to assess more recent willingness data from 2021 and to understand its predictors.

In many countries, age and gender were identified as significant predictors of vaccination willingness [e.g., 8, 9]. However, the direction of their effects varied between the countries. In China, younger age predicted more willingness [11, 19]. In contrast, older people were more likely to report vaccination willingness than younger ones in Poland, France, Germany, Sweden [19], the U.K. [13] and the U.S. [16]. Male persons showed a higher vaccination willingness in the U.S. [12], while the willingness was higher in female persons in France, Germany, Sweden [19] and Poland [20]. In Russia, Lazarus, Wyka [19] reported a higher willingness in female persons, while in another study male persons showed a higher willingness [17]. In Spain, married people revealed a higher willingness [18]. Lower income or social status served as a positive willingness predictor in China [11] and in Russia [17].

While the available investigations on willingness predictors mainly focused on demographics, further factors such as the experience of Covid-19 consequences, variables of mental health, evaluation of governmental actions, and the source of Covid-19 information have so far attracted little attention. The few available studies mostly focused on samples from one country. Thus, due to the lack of cross-national findings, it remains unclear and should investigated whether the results reveal universal predictors and whether the direction of their effects on vaccination willingness remains stable across different countries.

The present study belongs to the ongoing international “Bochum Optimism and Mental Health (BOOM)”-Project that investigates risk and protective factors of mental health. Using the framework of previous comparisons of countries with different welfare systems [21–24], the current focus was on the following nine countries: China (CH), France (FR), Germany (GE), Poland (PL), Russia (RU), Spain (ES), Sweden (SV), the U.K. (UK), and the U.S. (US). To close the described research gap, we had two main aims: 1.) to investigate the general population willingness for Covid-19 vaccination in the nine countries simultaneously and to compare it between the countries; 2.) to identify factors that could influence the vaccination willingness in the end of spring 2021 –after more than one year of living with the Covid-19 situation and its consequences for everyday life [25, 26]. We focused on five groups of potential predictors of the vaccination willingness.

First, based on previous research [e.g., 19], we focused on the demographics. In addition to gender, age, marital status, and social status, we added urbanicity as a potential predictor. In a recent study from India, people from urban areas showed a low vaccination willingness in March 2020 [27]. In Japan, the willingness was positively associated with living in rural areas in September 2020 [28].

Second, belonging to the Covid-19 risk group (e.g., pre-existing health conditions, age-related, weakened immune system), risk perception and perceived Covid-19 infectability served as positive willingness predictors in China [29], Iran [30], Spain [18] and Taiwan [31]. In Germany and Iran, the psychological burden caused by the Covid-19 situation and the anxiety of health-related consequences caused by the virus were identified as its positive predictors [15, 30, 32]. Furthermore, broadly formulated, vaccination willingness belongs to behavioral factors that are introduced to fight the pandemic. In a recent study, being affected in terms of physical and mental health, as well as positive mental health (PMH)–the social, emotional and psychological well-being [33]–positively predicted adherence to the governmental behavioral measures and factors [34]. In contrast, being affected economically by the Covid-19 situation and the level of experienced stress were its negative predictors [34, 35]. Against this background, we included the belonging to a Covid-19 risk group, the level of experienced physical, mental, and economic consequences of the Covid-19 outbreak, the level of depression, anxiety, and stress symptoms, psychological burden caused by the Covid-19 situation and PMH as potential predictors of the Covid-19 vaccination willingness.

Third, available research shows that being well-informed about the vaccines, herd immunity and further factors that are linked to the pandemic is an important predictor of the willingness for Covid-19 vaccination [14, 36, 37]. While the traditional media such as television reports and newspaper reports (print media) as well as official governmental online sites allow a passive consumption of filtered information, social media such as Facebook and Twitter provide the users with a permanent access to new unfiltered information and enable an active participation in the creation, modification and sharing of the content [38]. This, however, enhances the risk of a rapid spread of misinformation and fake news amplified by emotional comments [39–41] that can significantly impact the users’ evaluation of the Covid-19 situation and their behavior [42]. In Germany, individuals who used more official governmental sites than social media to stay up-to-date about the Covid-19 situation showed a higher vaccination willingness [15]. In the U.K., the use of social media as a Covid-19 information source was negatively linked to the willingness [43]. Against this background, we focused on the source of Covid-19 information (i.e., television reports, print media, official governmental sites, and social media) as potential predictors of the Covid-19 vaccination willingness.

Fourth, in a study that investigated different Asian countries such as China and Vietnam and in a study that focused on Russia, the trust in the health care system predicted the vaccination willingness positively [17, 44]. Furthermore, in a cross-national study, the evaluation of the governmental communication as for example credible and honest or guided by the interests of people was positively linked to the adherence to Covid-19 measures. In contrast, individuals who reported that they feel left alone by the government showed less adherence [34]. Thus, we included the evaluation of the governmental communication about the Covid-19 situation (i.e., as clear and understandable, credible and honest, guided by interests of people) and how the population perceives to be treated by the government and authorities (i.e., well supported, well informed, taken seriously, left alone) since the pandemic outbreak as potential predictors of the Covid-19 vaccination willingness.

Fifth, we included the usefulness evaluation of the Covid-19 measures and the general adherence to the measures as further potential predictors of the vaccination willingness.

## Methods

### Procedure and participants

The overall investigated sample included 9,264 participants from nine countries: CH: *N* = 1,020; FR: *N* = 1,001, GE: *N* = 1,145, PL: *N* = 1,004, RU: *N* = 1,024, ES: *N* = 985, SV: *N* = 1,003, UK: *N* = 1,050, and US: *N* = 1,032. Table 1 presents the country-specific demographics. An independent social marketing and research institute (YouGov, www.yougov.de) collected the data via population-based online-panel surveys in the national language of the countries within eight days (May 12 to May 19, 2021). Participants were recruited from residential populations aged 18 years and above. YouGov implemented age, gender, and region stratification to achieve representativeness. Participants were compensated by panel-specific tokens that could be converted in voucher or monetary payment. The response rate was: 92.5% in China, 91.9% in France, 94.5% in Germany, 90.3% in Poland, 92.3% in Russia, 88.7% in Spain, 90.5% in Sweden, 91.1% in the U.K., and 86.4% in the U.S. The responsible Ethics Committee approved our study’s implementation (approval number: 118 extended). YouGov obtained the required permits and approvals for the data collection in all nine countries. The study was pre-registered with AsPredicted.org on May 05, 2021 (Pre-registration Number: #64865). All participants were properly instructed and gave online their informed consent to participate via an online form. All national regulations and laws regarding human subjects research were followed. Power analyses (G*Power program, version 3.1) revealed that the sample sizes are sufficient for valid results (power >.80, *α* = .05, effect size: *f*^*2*^ = .15; [cf., 45]). The dataset used in the present study is available in S1 Dataset.

**Table 1:** Country-specific demographic variables.

	CH	FR	GE	PL	RU	ES	SV	UK	US
**Gender (female, %)**	44.2	54.9	52.0	53.7	51.5	51.9	51.5	55.4	51.3
**Age groups (%)**									
**18 to 24 years**	25.4	8.8	7.7	9.7	8.5	7.5	6.0	2.7	8.4
**25 to 34 years**	35.5	14.8	14.2	18.1	22.0	14.1	19.6	13.4	13.5
**35 to 44 years**	23.4	16.3	14.5	19.7	21.6	19.7	12.7	15.0	15.0
**45 to 54 years**	11.4	17.4	19.8	15.6	18.2	20.4	17.1	15.7	18.2
**55 years and older**	4.3	42.8	43.8	36.9	29.8	38.3	44.6	53.2	44.9
**Marital status (with partner, %)**	56.3	62.3	57.6	64.5	70.0	64.1	55.3	64.5	61.3
**Social Status (%)**									
**Lower class**	16.9	7.7	6.9	4.8	4.4	4.1	6.9	3.0	8.7
**Working class**	49.8	20.6	18.9	17.7	17.5	33.9	21.8	32.6	17.4
**Lower middle class**	17.4	31.0	28.7	34.0	36.1	20.1	14.8	26.8	18.8
**Middle middle class**	12.6	30.9	36.9	32.5	35.4	36.8	42.9	32.7	35.9
**Upper middle class**	2.6	8.7	7.7	8.5	5.0	5.1	12.8	4.6	16.2
**Upper class**	0.7	1.2	1.0	2.6	1.6	0.1	0.8	0.4	2.9
**Urbanicity (large city, %)**	42.9	29.9	35.2	43.8	74.7	38.6	45.4	22.3	35.9

China (CH): *N* = 1,020, France (FR): *N* = 1,001, Germany (GE): *N* = 1,145, Poland (PL): *N* = 1,004, Russia (RU): *N* = 1,024, Spain (ES): *N* = 985, Sweden (SV): *N* = 1,003, the U.K. (UK): *N* = 1,050, the U.S. (US): *N* = 1,032; due to rounding, the sum of the frequencies is not always 100%.

### Measures

#### Demographics

Following previous research on population samples (e.g., [34, 46]), participants were asked to indicate their gender (0 = *woman*, 1 = *man*), age range (1 = *18 to 24 years*, 5 = *55 years and older*), marital status (0 = *without partner*, 1 = *with partner*), social status (1 = *lower class*, 6 = *upper class*), and urbanicity (0 = *small city or rural community*, 1 = *large city*) (see Table 1 for details).

#### Covid-19 specific content

To assess Covid-19 vaccination willingness, participants were asked to answer the question “Have you already been vaccinated against Covid-19 at least once?” using the three options: 1 = *No*, *and I do not want to be vaccinated*, 2 = *No*, *but I would like to be vaccinated*, 3 = *Yes*. This question was formulated for the present study. We discussed its wording with experts in health research and medicine. To calculate willingness, the ratings “2” and “3” were merged to yield a binary variable: 0 = *No*, *and I do not want to be vaccinated*, 1 = *Yes* and *No*, *but I would like to be vaccinated*.

Furthermore, following Margraf, Brailovskaia [34] and Brailovskaia, Cosci [47], participants were asked to rate 1) whether they belonged to the Covid-19 risk group (0 = *no*, 1 = *yes*); 2) to what extent they were affected by the Covid-19 situation 2a) in terms of physical health, 2b) economically, and 2c) mentally, respectively, on a 5-point Likert-type scale (0 = *not at all*, 4 = *very strong*); 3) their usage frequency of 3a) news reports on television, 3b) newspaper articles (print media), 3c) official sites of the national government and authorities, and 3d) social media (e.g., Twitter, Facebook) as a Covid-19 information source, respectively, on a 7-point Likert-type scale (1 = *not at all*, 7 = *very intensively*); 4) to what extent they assessed the communication of the national government and authorities regarding the Covid-19 situation as 4a) clear and understandable, 4b) credible and honest, and 4c) guided by the interests of the people, respectively, on a 5-point Likert-type scale (1 = *not at all true*, 5 = *very true*); 5) to what extent they felt 5a) well supported, 5b) well informed, 5c) taken seriously, and 5d) left alone by the national government and authorities, respectively, on a 5-point Likert-type scale (1 = *not at all true*, 5 = *very true*); 6) to what extent they considered the introduced measures to combat the Covid-19 crisis as useful and 7) how much they adhered to the measures, respectively, on a 5-point Likert-type scale (0 = *not at all*, 4 = *very strong*). The rating of the governmental communication was not assessed in China.

#### Depression, anxiety, and stress symptoms

The Depression Anxiety Stress Scales 21 (DASS-21; [48]) assessed symptoms of depression, anxiety and stress with, respectively, seven items per subscale (e.g., depression subscale: “I felt that life was meaningless”; anxiety subscale: “I felt scared without any good reason”; stress subscale: “I found it hard to wind down”). The items are rated on a 4-point Likert-type scale (0 = *did not apply to me at all*, 3 = *applies to me very much or most of the time*). The higher the sum scores, the higher the negative symptoms. In the present study, internal consistency scores for the subscales ranged from *α* = .899 (CH) to .941 (UK) for depression, *α* = .862 (GE) to .901 (US) for anxiety, and *α* = .877 (CH) to .919 (GE, PL) for stress.

#### Psychological burden caused by Covid-19

The Covid-19 Burden Scale [49] assessed the psychological burden caused by the Covid-19 situation. The six items (e.g., “I am burdened by the current social situation”) are rated on a 7-point Likert-type scale (1 = *I do not agree*, 7 = *I totally agree*). Higher sum scores indicate higher levels of burden. In the present study, internal consistency scores ranged from *α* = .665 (US) to *α* = .791 (GE) with exception of China (*α* = .347).

#### Positive Mental Health (PMH)

PMH was measured with the unidimensional Positive Mental Health Scale (PMH-Scale; [33]). The PMH-Scale is an internationally well-established instrument for the assessment of psychological, emotional, and social well-being [50]. The nine items are rated on a 4-point Likert-type scale (e.g., “I enjoy my life”; 0 = *do not agree*, 3 = *agree*). Higher sum scores indicate higher levels of PMH. In the present study, internal consistency scores ranged from *α* = .896 (FR) to .937 (SV).

Previously validated national language versions of the included instruments were used (e.g., PMH: [34, 51]; DASS-21: [52]). In case that no previously validated national language version was available, the international team of the BOOM-Project translated the scales into the national language from the English language version by the customary translation-back-translation-modification procedure [53].

### Statistical analyses

Statistical analyses were conducted using the Statistical Package for the Social Sciences (SPSS 26; [54]). First, descriptive statistics of the investigated variables were calculated for all national samples. Next, willingness for Covid-19 vaccination (0 = *No*, *and I do not want to be vaccinated*, 1 = *Yes* and *No*, *but I would like to be vaccinated*) was compared between the nine countries via Pearson Chi-Square tests. Cramer’s V served as the effect size measure [55]. The comparisons were all Bonferroni-corrected (level of significance: *p* < .05, two-tailed). Then, a logistic regression analysis was computed in each country to examine the contribution of potential predictors of Covid-19 vaccination willingness. The analysis consisted of two steps. To control for demographic variables (gender, age group, marital status, social status, urbanicity), these were included in Step 1. Step 2 then included the variables belonging to the risk group; being affected by the Covid-19 situation in terms of physical health, economically and mentally; symptoms of depression, anxiety and stress; psychological burden by Covid-19; PMH; the use of different Covid-19 information sources (television reports, print media, official sites, social media); perception of the governmental communication as clear and understandable, credible and honest, guided by interests of people; the feeling of being well supported, well informed, taken seriously or left alone by the government; evaluation of the Covid-19 measures as useful; and the adherence to the measures. The calculated odds ratios (OR) for each predictor variable are presented.

## Results

Table 2 presents the descriptive statistics of the investigated variables in the nine country-specific samples.

**Table 2:** Country-specific descriptive statistics of the investigated variables.

	CH	FR	GE	PL	RU	ES	SV	UK	US
**Covid-19 vaccination willingness (%)**	86.9	68.9	81.7	71.3	62.0	89.6	83.8	93.9	80.6
**Risk group (yes, %)**	4.8	26.0	40.3	38.7	46.4	27.8	36.3	38.8	46.7
**M (SD)**									
**Affected: Health**	.87(1.00)	1.42(1.19)	.84(1.07)	1.40(1.18)	1.80(1.31)	1.51(1.15)	1.24(1.16)	.64(.99)	.91(1.15)
**Affected: Economically**	1.34(1.10)	1.35(1.22)	1.08(1.16)	1.82(1.21)	2.06(1.29)	1.48(1.25)	.90(1.14)	.85(1.10)	1.30(1.25)
**Affected: Mentally**	1.43(1.08)	1.50(1.15)	1.56(1.18)	1.84(1.22)	1.47(1.27)	1.74(1.11)	1.66(1.23)	1.36(1.15)	1.53(1.24)
**Depression Symptoms**	5.42(4.94)	4.72(5.40)	4.97(5.30)	6.54(5.67)	5.76(4.99)	6.09(5.66)	4.86(5.45)	5.09(5.44)	5.20(5.66)
**Anxiety Symptoms**	5.26(4.44)	3.68(4.56)	3.18(4.03)	5.20(5.01)	4.59(4.80)	4.64(5.02)	3.40(4.45)	2.87(3.88)	3.93(4.89)
**Stress Symptoms**	6.34(4.62)	5.60(5.36)	5.50(5.15)	7.07(5.43)	6.70(5.12)	7.12(5.24)	5.52(4.83)	5.24(4.96)	5.41(5.19)
**Burden by Covid-19**	3.30(.87)	4.35(1.17)	3.85(1.26)	3.93(1.22)	4.04(1.28)	4.50(1.13)	3.65(1.19)	3.23(1.11)	3.26(1.14)
**Positive mental health**	16.65(5.93)	16.23(5.45)	16.68(5.94)	17.04(6.66)	16.36(5.48)	16.83(5.69)	16.14(6.85)	16.49(5.81)	17.92(6.00)
**Information Source**									
**Television Reports**	4.21(1.97)	4.12(1.86)	4.67(1.99)	4.19(2.19)	4.51(2.22)	4.84(1.88)	4.10(1.95)	4.09(1.91)	3.92(2.11)
**Print Media**	2.49(1.73)	2.62(1.76)	3.32(2.11)	2.69(1.86)	2.61(1.91)	2.96(1.98)	2.79(1.91)	2.42(1.83)	2.84(1.98)
**Official Sites**	4.14(1.86)	3.06(1.81)	3.63(1.96)	3.26(1.95)	3.59(2.13)	3.45(1.99)	3.40(1.82)	3.41(1.86)	3.70(1.99)
**Social Media**	5.06(1.67)	2.87(1.89)	2.72(1.92)	3.91(2.00)	3.79(2.12)	3.88(2.04)	2.96(1.86)	2.47(1.72)	3.03(1.98)
**Communication (government, authorities)**									
**Clear & understandable**		2.28(1.14)	2.62(1.19)	2.62(1.25)	3.18(1.32)	2.45(1.19)	2.84(1.33)	2.98(1.27)	3.02(1.28)
**Credible & honest**		2.24(1.16)	2.60(1.22)	2.35(1.26)	2.79(1.32)	2.36(1.19)	2.83(1.34)	2.73(1.32)	2.95(1.36)
**Guided by interests of people**		2.41(1.20)	2.41(1.16)	2.31(1.24)	2.76(1.34)	2.29(1.17)	2.56(1.23)	2.73(1.31)	2.94(1.36)
**Feeling of being … by government, authorities**									
**…well supported…**	3.96(1.14)	2.31(1.14)	2.51(1.12)	2.14(1.15)	2.59(1.31)	2.25(1.12)	2.38(1.18)	2.94(1.19)	2.98(1.30)
**…well informed…**	4.00(1.07)	2.44(1.14)	2.79(1.20)	2.48(1.21)	3.11(1.29)	2.44(1.17)	3.04(1.30)	3.09(1.20)	3.11(1.31)
**…taken seriously…**	4.15(1.02)	2.35(1.16)	2.54(1.21)	2.26(1.22)	2.91(1.27)	2.32(1.15)	2.58(1.21)	3.04(1.23)	3.20(1.31)
**…left alone…**	1.85(1.06)	2.86(1.33)	3.10(1.32)	3.17(1.37)	2.68(1.34)	3.00(1.33)	2.75(1.29)	2.60(1.17)	2.40(1.21)
**Measures Usefulness**	2.67(1.17)	1.79(1.12)	2.35(1.26)	1.91(1.20)	2.31(1.21)	2.23(1.17)	2.06(1.17)	2.67(1.05)	2.32(1.37)
**Adherence to Measures**	2.89(1.12)	2.97(1.00)	3.06(1.02)	2.68(1.16)	2.41(1.08)	3.17(.93)	2.87(1.03)	3.22(.91)	2.83(1.24)

China (CH): *N* = 1,020, France (FR): *N* = 1,001, Germany (GE): *N* = 1,145, Poland (PL): *N* = 1,004, Russia (RU): *N* = 1,024, Spain (ES): *N* = 985, Sweden (SV): *N* = 1,003, the U.K. (UK): *N* = 1,050, the U.S. (US): *N* = 1,032; *M* = Mean; *SD* = Standard Deviation.

**Fig 1:**
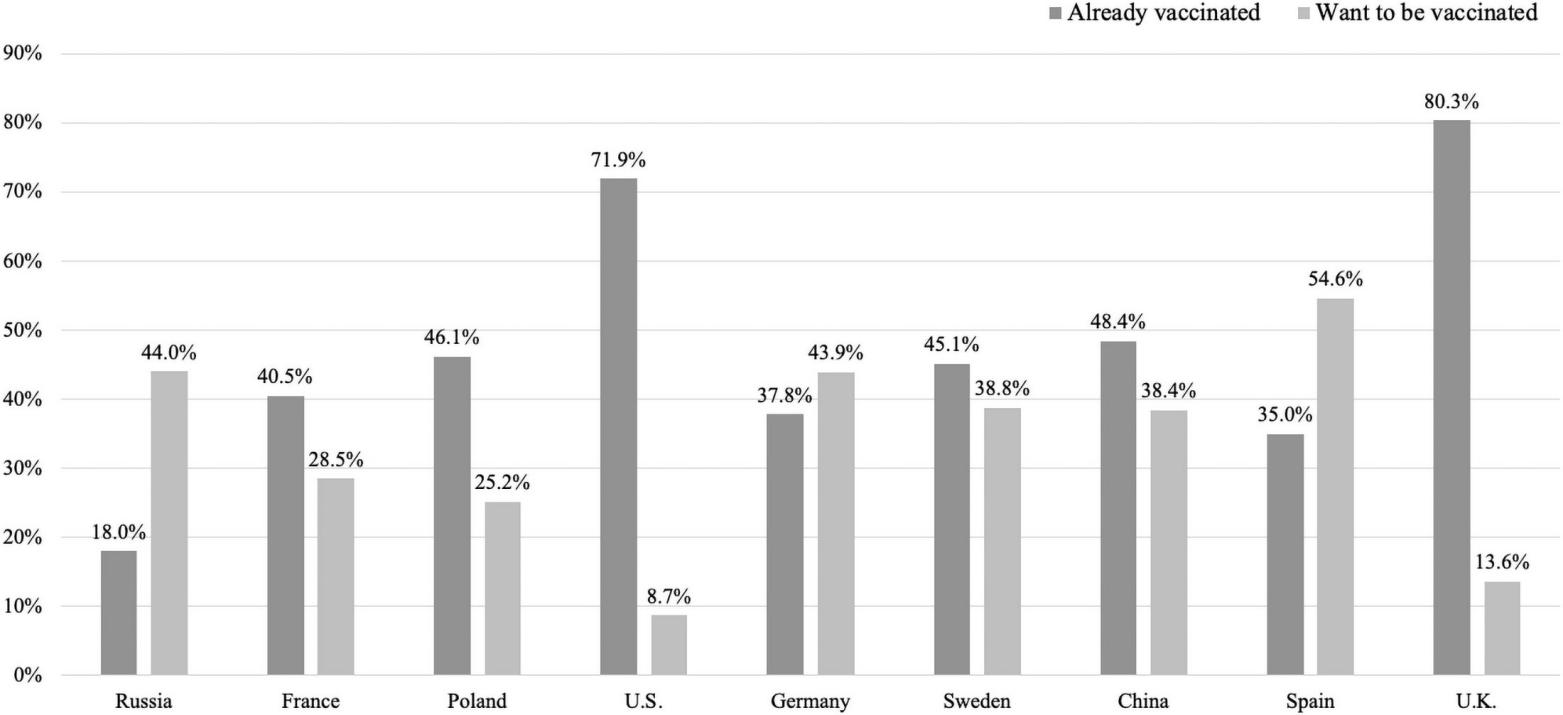
Percentage of participants who are already Covid-19 vaccinated or who want to be vaccinated (country-specific samples). Notes. China: N = 1,020, France: N = 1,001, Germany: N = 1,145, Poland: N = 1,004, Russia: N = 1,024, Spain: N = 985, Sweden: N = 1,003, the U.K.: N = 1,050, the U.S.: N = 1,032.

Overall, 7,405 (79.9%) participants revealed willingness for Covid-19 vaccination: 4,431 (47.8%) were already vaccinated and 2,974 (32.1%) wanted to be vaccinated. The highest willingness was in the U.K. with 93.9%, followed by Spain, China, Sweden, Germany, the U.S., Poland, and France. The lowest willingness was in Russia with 62% (see Table 2). Fig 1 visualizes the rate of participants who were already vaccinated and those who wanted to be vaccinated but were not yet vaccinated at the time of data collection. The U.K. showed the highest proportion of already vaccinated persons (80.3%), followed by the U.S., China, Poland, Sweden, France, Germany, and Spain. Russia showed the lowest rate of vaccinated participants (18%). The rate of participants who wanted to be vaccinated but were still not vaccinated was highest in Spain (54.6%), followed by Russia, Germany, Sweden, China, France, Poland, and the U.K. The lowest rate was in the U.S. (8.7%) (see Fig 1).

**Fig 2:**
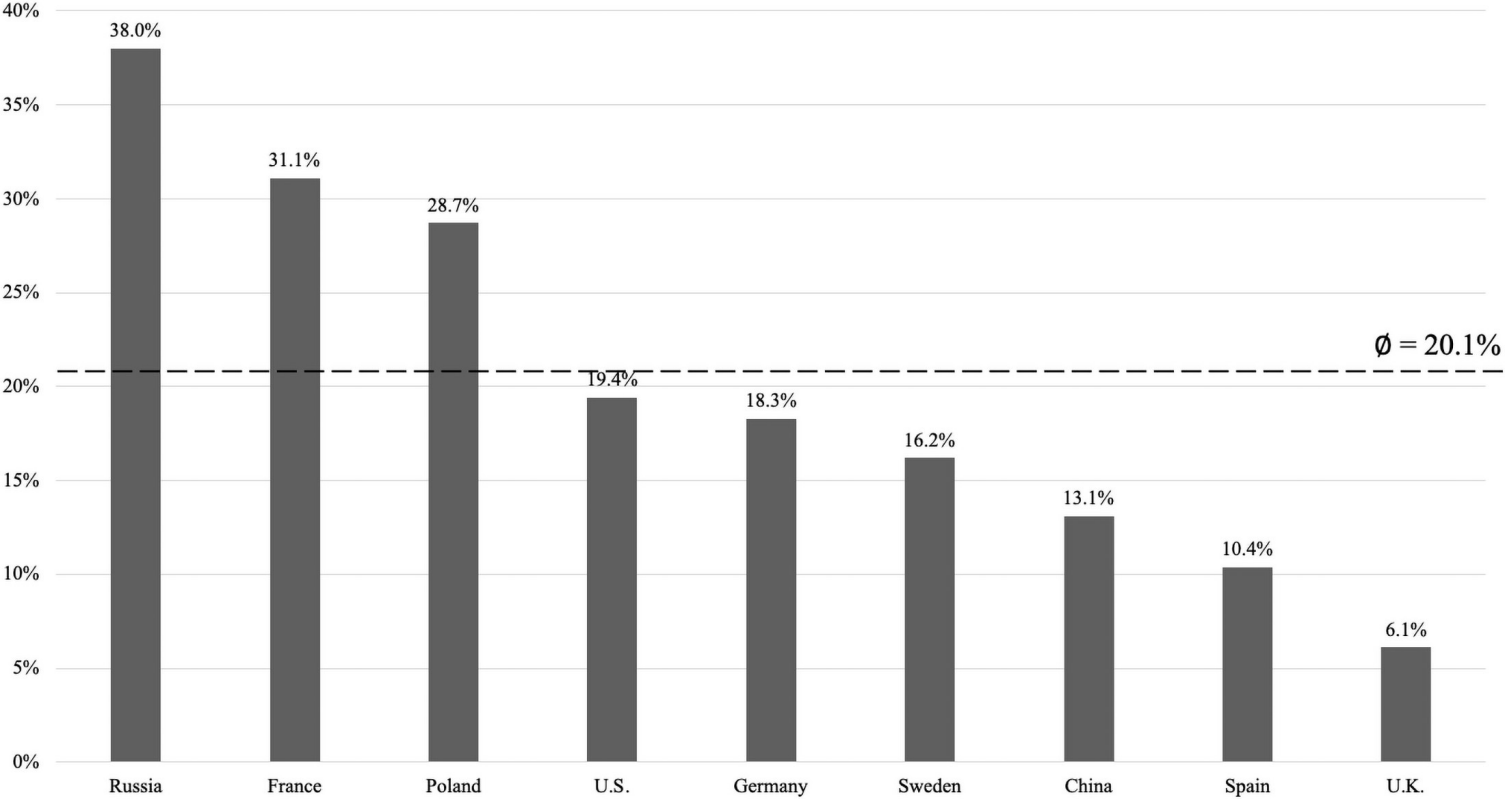
Percentage of participants who do not want to be Covid-19 vaccinated (country-specific samples). Notes. China: N = 1,020, France: N = 1,001, Germany: N = 1,145, Poland: N = 1,004, Russia: N = 1,024, Spain: N = 985, Sweden: N = 1,003, the U.K.: N = 1,050, the U.S.: N = 1,032. Overall vaccination rejection = 20.1%.

Overall, 1,859 (20.1%) participants did not want to be vaccinated. Fig 2 visualizes the rate of vaccination refusal in the country-specific samples. Notably, in Russia, France and Poland, the rejection rate was higher than 20.1% (i.e., overall rejection rate). The difference of 31.9% between Russia, the country with the highest rejection rate (38%), and the U.K., the country with the lowest rejection rate (6.1%), was remarkably high.

The Pearson Chi-Square tests revealed significant differences between the investigated countries in terms of vaccination willingness. Table 3 provides the effect sizes of the significant differences that ranged between small and medium. Of the 36 comparisons only six were not significant. The U.K. was the country with the significantly highest willingness for Covid-19 vaccination in comparison to the other eight included countries (significant differences: UK > CH, FR, GE, PL, RU, ES, SV, US). Spain had the second highest willingness rate (significant differences: ES > FR, GE, PL, RU, SV, US), followed by China (significant differences: CH > FR, GE, PL, RU, US). In contrast, Russia was the country with the significantly lowest vaccination willingness (significant differences: RU < CH, FR, GE, PL, ES, SV, UK, US), followed by France (significant differences: FR < CH, GE, ES, SV, UK, US) and Poland (significant differences: PL < CH, GE, ES, SV, UK, US) (see Table 1: percentage of willingness, and Table 3: effect size of the Pearson Chi-Square test).

**Table 3:** Simplified presentation of the effect sizes (Cramer’s V) of the comparison of Covid-19 vaccination willingness between the countries via Pearson Chi-Square test.

	**Effect Size Cramer’s V of Significant Differences**							
	FR	GE	PL	RU	ES	SV	UK	US
**CH**	.216	.070	.191	.285	n.s.	n.s.	.120	.085
**FR**		.149	n.s.	.073	.255	.176	.323	.135
**GE**			.123	.220	.112	n.s.	.184	n.s.
**PL**				.099	.231	.150	.300	.109
**RU**					.321	.245	.386	.206
**ES**						.085	.078	.126
**SV**							.161	n.s.
**UK**								.200
**US**								

China (CH): *N* = 1,020, France (FR): *N* = 1,001, Germany (GE): *N* = 1,145, Poland (PL): *N* = 1,004, Russia (RU): *N* = 1,024, Spain (ES): *N* = 985, Sweden (SV): *N* = 1,003, the U.K. (UK): *N* = 1,050, the U.S. (US): *N* = 1,032; Cramer’s V is presented for significant comparisons; n.s. = not significant comparison; interpretation of Cramer’s V: 0.1 ≤ Cramer’s V < 0.3: small effect, 0.3 ≤ Cramer’s V < 0.5: medium effect, Cramer’s V > 0.5: large effect [55].

As shown in Table 4, the logistic regression analyses yielded significant results in all investigated countries. Overall, the explained variance for the Covid-19 vaccination willingness ranged roughly between 30% and 40% with the notable exception of China with only 12.9%. However, there were some noteworthy differences between countries in the direction of the effects and in the significant predictors.

**Table 4:** Country-specific logistic regression analyses (outcome: Covid-19 vaccination willingness; 0 = no willingness, 1 = willingness).

	Odds Ratio								
	CH	FR	GE	PL	RU	ES	SV	UK	US
**Step 1**									
**Gender**	**1.597** *	**.661** *	**.526** **	.722	**.417** **	.778	.795	.539	**.586** *
**Age Group**	**.791** *	**1.317** **	1.107	1.094	.986	.985	1.140	1.229	.833
**Marital Status**	**.608** *	1.108	1.260	1.092	**.640** **	**.493** **	.684	**.530** *	1.057
**Social Status**	1.121	**1.315** **	1.083	1.134	1.164	.816	**1.310** **	1.367	**1.344** **
**Urbanicity**	1.024	.734	.980	**.544** **	**.705** *	1.185	.954	.502	.676
**Step 2**									
**Risk Group**	1.168	**3.182** **	1.488	1.134	1.339	1.811	**2.000** **	1.596	**1.929** **
**Affected: Health**	1.154	**1.223** *	1.040	1.141	**1.208** *	.889	**.755** *	1.095	**.801** *
**Affected: Economically**	.907	**.802** **	.949	.955	.896	.841	.948	.824	**.801** *
**Affected: Mentally**	1.008	1.184	1.086	1.176	.976	1.130	**1.536** **	.912	1.024
**Depression Symptoms**	1.070	1.044	1.036	1.017	1.031	1.059	.992	.962	1.010
**Anxiety Symptoms**	.964	.955	.992	.976	1.008	.962	1.029	1.114	1.055
**Stress Symptoms**	1.015	1.013	**.913** *	.958	.994	1.021	.962	.991	**.909** *
**Psychological Burden by Covid-19**	.766	.948	1.131	1.102	.967	1.066	1.076	1.084	.891
**Positive Mental Health**	**1.047** *	1.010	**.957** *	.991	1.017	1.023	1.001	.971	.921**
**Info-Source: TV Report**	.983	**1.165** **	**1.163** **	1.061	**1.112** *	**1.216** **	**1.328** **	**1.298** *	1.075
**Info-Source: Print Media**	1.041	1.083	1.065	**1.246** **	1.069	1.038	1.059	.996	1.078
**Info-Source: Official Sites**	**1.285** **	1.066	**1.147** *	1.065	1.015	.893	1.128	1.183	1.053
**Info-Source: SM**	.937	.924	.990	**.886** **	1.014	.919	**.862** *	1.018	**.861** *
**Commun.: Clear & Understandable**		.964	.752	.920	.851	1.172	**.720** *	1.006	**.744** *
**Commun.: Credible & Honest**		.980	1.175	.920	1.136	.917	**1.796** **	1.402	**1.489** *
**Commun.: Guided by people’s interests**		.989	1.287	**1.373** *	1.205	1.215	.843	.775	1.201
**Feel: …well supported**	1.129	1.130	.790	**1.326** *	.863	1.012	.941	1.179	1.098
**Feel: …well informed**	1.239	1.144	1.224	1.161	1.156	.785	.903	1.203	1.035
**Feel: …taken seriously**	.858	1.027	1.178	**.592** **	1.235	1.254	1.312	.814	**1.301** *
**Feel: …left alone**	.910	**.824** **	.913	1.041	.991	.867	1.046	1.018	.944
**Measures Usefulness**	.960	**1.480** **	**2.237** **	**1.535** **	**1.418** **	**2.006** **	**1.730** **	**2.049** **	**1.914** **
**Adherence to Measures**	1.015	.968	1.026	**1.546** **	**1.434** **	**1.512** **	**1.286** *	**1.432** *	1.191
**Nagelkerke R** ^**2**^	**.*129***	**.*351***	**.*410***	**.*360***	**.*375***	**.*290***	**.*390***	**.*379***	**.*453***

China (CH): *N* = 1,020, France (FR): *N* = 1,001, Germany (GE): *N* = 1,145, Poland (PL): *N* = 1,004, Russia (RU): *N* = 1,024, Spain (ES): *N* = 985, Sweden (SV): *N* = 1,003, the U.K. (UK): *N* = 1,050, the U.S. (US): *N* = 1,032; SM = Social Media; Commun. = Governmental Communication; Marital Status: reference “with partner”; Urbanicity: reference “large city”; Gender: 0 = *woman*, 1 = *man* (reference);

**p < .01,

*p < .05.

To sum up, of the 27 included potential predictors, twelve showed significant results in the U.S., ten in Sweden, nine in France, eight in Poland, seven in Russia, six in Germany, five in China, and respectively four in Spain and the U.K. In most countries, the perception of introduced measures to fight Covid-19 as useful was a significant predictor (eight of nine countries), followed by the use of television reports as Covid-19 information source (six of nine countries) (see Table 4).

More specifically, in Step 1, gender was a significant predictor in five countries (CH: men > women; FR, GE, RU, US: women > men). Age group was a significant predictor in China (younger people > older people) and in France (older people < younger people). Marital status was a significant predictor in four countries (CH, RU, ES, UK: without partner > with partner). Social status was a significant predictor in three countries (FR, SV, US: higher status > lower status). Urbanicity served as a significant predictor in Poland and Russia (both: small city or rural community > large city) (see Table 4).

In Step 2, belonging to the Covid-19 risk group was a significant positive predictor in three countries (FR, SV, US). Being affected in terms of physical health served as a significant positive predictor in France and Russia, but as a significant negative predictor in Sweden and the U.S. Being affected economically was a significant negative predictor in France and the U.S. Being affected mentally was a significant positive predictor in Sweden (see Table 4).

Considering the mental health factors, depression, anxiety, and psychological burden showed no significant results. Stress symptoms served as a significant negative predictor in Germany and the U.S. Remarkably, PMH was a significant positive predictor in China, but a significant negative predictor in Germany and the U.S. (see Table 4).

As shown in Table 4, the source of Covid-19 information yielded significant results. The use of television reports was a significant positive predictor in six countries (FR, GE, RU, ES, SV, UK); the use of print media served as a significant positive predictor in Poland; the use of official sites was a significant positive predictor in China and Germany; in contrast, the use of social media was a significant negative predictor in three countries (PL, SV, US).

The perception of governmental communication as clear and understandable was a significant negative predictor and as credible and honest was a significant positive predictor in Sweden and the U.S.; its perception as guided by people’s interests was a significant positive predictor in Poland (see Table 4).

The feeling of being well supported served as a significant positive predictor in Poland; the feeling of being well informed showed no significant results; the feeling of being taken seriously was a significant negative predictor in Poland and a significant positive predictor in the U.S.; and the feeling of being left alone was a significant negative predictor in France (see Table 4).

Finally, the perceived usefulness of the Covid-19 measures served as a significant positive predictor in eight of the nine countries (exception: CH). And the adherence to the measures was a significant positive predictor in five countries (PL, RU, ES, SV, UK) (see Table 4).

## Discussion

Over one year, research teams around the globe such as those involved in the “Project Lightspeed” [1] have been working on vaccines to combat the Covid-19. The success of their work largely depends on the willingness for vaccination among the population [8]. Therefore, it is of great importance to identify modifiable factors that can predict the willingness.

The present study provides representative results on vaccination willingness and its potential predictors from nine different countries on three continents. Overall, and in six of the nine individual national samples (exceptions: RU 62%, FR 68.9%, PL 71.3%), about four-fifths of the participants or even more reported vaccination willingness in spring 2021. Considering that about 71–74% of the population should be vaccinated to achieve herd immunity [see 8], the results are encouraging. Furthermore, in eight of the nine investigated countries, the level of willingness was remarkable higher than the level that was found by Lazarus, Ratzan [9] in June 2020. Only in China, we found a slightly lower willingness level (difference: about 1.7%) that however was higher than the figures reported by Chen, Li [10] and Liu, Zhang [11] in 2020.

Notably, the current willingness in Russia was higher than the one reported by Lazarus, Ratzan [9] and Tran, Pak [17] in 2020. However, it was considerably below the required threshold [see 8] which is a cause of concern and worry—given that Russia belongs to the ten countries with the worldwide largest population [56]. The vaccination willingness in Russia was significantly lower than the willingness in the other eight investigated countries. Of the overall 36 comparisons, the highest significant difference (medium effect size) was between Russia and the U.K.

The U.K. was the country with the highest vaccination willingness in comparison to the other included countries and with the highest increase of the willingness (22.4%) compared to the figures assessed in 2020 by Lazarus, Ratzan [9]. The present results complement the findings of Margraf, Brailovskaia [34] who found the highest adherence to governmental measures in the U.K. and the lowest in Russia in summer 2020. Spain showed the second highest vaccination willingness in the current study and the second highest adherence in Margraf, Brailovskaia [4]; Poland had a lower vaccination willingness than six of the other investigated countries in 2021 and also a relatively low adherence level in 2020 [34]. However, the result patterns were different in France: While Margraf, Brailovskaia [34] reported a relatively high level of adherence to the governmental measures in 2020 in France, the vaccination willingness in this country was relatively low in 2020 [9] and it remained also relatively low in 2021. In our study, willingness was lower in France than in six of the other investigated countries. Thus, it seems that measures that do not refer to invasive interventions are evaluated differently in France than these that include invasive steps such as vaccination. Invasive interventions might be linked to more safety concerns than for example the wearing of face masks or the keeping of distance [57], and thus could meet a higher level of rejection in France. The vaccination willingness in Germany (difference between 2021 and 2020: about 13%) and Sweden (difference between 2021 and 2020: about 19%) was higher in the present study than in the work of Lazarus, Ratzan [9]. However, in both studies, these countries had a “middle field” position in the ranking of the nine focused countries. In 2020, the willingness was the second highest in the U.S. in comparisons to the other eight countries [9]. In 2021, the U.S. had the sixth place in the ranking.

Our regression results reveal that the differences of vaccination willingness between the nine countries could partly be explained by a range of internal and external predictors. Some of them—such as the evaluation of governmental communication—were rather specific for only a few countries. Others—such as the evaluation of the introduced measures as useful—had an almost universal effect on willingness. Moreover, some predictors—such as age and gender, but also PMH—showed differences in the direction of the effects between countries which could indicate a substantial influence of sociocultural factors. Overall, the included predictors explained about 30% to 40% of the variance. The country with the highest explained variance was the U.S., followed by Germany and Sweden. Only in China, the proportion was remarkably lower (below 13%). This might partly be due to the reduced number of predictors assessed in China. However, this difference might also refer to specific characteristics of this country. Note that the direction of some significant effects (e.g., gender) differed between China and some of the other investigated countries.

The present findings confirm previous research that described demographic factors as potential predictors for willingness [e.g., 16, 19, 58]. In line with earlier results [11, 19], younger people reported significantly more vaccination willingness in China. We hypothesize that younger people in China might suffer more from the governmental restrictions on gathering and traveling than older ones, especially because many of them study abroad or work far away from their families [59, 60]. Therefore, they might be more willing for vaccination to gain back some freedom of mobility. Moreover, the willingness to protect others by getting oneself vaccinated was especially high in Asian countries [61]. In addition, concerns of vaccination safety were remarkably high in older people in China [11]. In contrast, in France age was positively related to vaccination willingness in the current study. This finding corresponds to the results from 2020 that focused directly on vaccination willingness [19] or on general adherence to the governmental measures [34] in France and other European countries. The enhanced perception of Covid-19 health-risk specifically in older people could partly explain this result [62]. Notably, in many European countries, the first vaccination campaigns focused specifically on older people and emphasized their high Covid-19 risk [63].

While in China male persons showed more willingness, female persons were more willing to accept vaccination in France, Germany, Russia, and the U.S. This finding is in line with earlier research that described gender to be a significant predictor of vaccination willingness. However, the direction of its effect significantly varied between and within countries [17, 19]. On the one hand, male gender is positively linked to risky behavior and sensation seeking [64] which might contribute to lower vaccination willingness. On the other hand, men are often responsible for the financial support of their family and therefore do not have the possibility to stay at home for a longer period of time [65] which could increase the wish for a vaccination. Female gender is positively associated with the support and care of others [66]. The wish to protect others could contribute to vaccination willingness [67]. In contrast, increased worries about potential side effects of the vaccination that are often higher in women than men might reduce it [32, 68].

Interestingly, in China, Russia, Spain, and the U.K., singles were more willing to be vaccinated than people with a partner. This contradicts recent research that described a higher willingness in married people [18, 69]. It might be that the higher vaccination willingness of singles is partly due to their wish to engage in offline dating that is limited by the measures (i.e., social distance) introduced to reduce the Covid-19 spread.

Furthermore, higher social status positively predicted vaccination willingness in France, Sweden, and the U.S. This is in line with a recent study from Japan indicating that that individuals with a higher social status rely stronger on the effectiveness of the Covid-19 vaccine than others [61]. However, this is not a universal explanation because in previous studies from China and Russia lower social status was positively associated with vaccination willingness [11, 17]. In both countries, health care services are often fee-based and expensive [70–72]. This could foster the vaccination willingness especially in people with a low social status and income who want to prevent negative health-related Covid-19 consequences that might cause further monetary costs.

In line with earlier findings from India and Japan [27, 28], individuals in rural communities or small cities in Poland and Russia were more likely to be vaccinated. This could be due to the lower availability of health care services in rural areas in comparisons to large cities and thus to more concerns about negative Covid-19 consequences. Furthermore, it could be that residents of large cities have a lower level of trust in the effectiveness of the Covid-19 vaccine and the governmental measures.

Beyond the demographic variables, we focused on potential consequences of Covid-19 and health related variables as potential predictors. In correspondence with available literature, belonging to the Covid-19 risk group was a positive predictor of vaccination willingness in France, Sweden, and the U.S. [18, 62, 73]. The predictive effect of being affected in terms of physical health showed varying directions (positive in FR and RU, negative in SV and US). Being affected economically was negatively linked to willingness in France and the U.S. Being affected mentally positively predicted willingness in Sweden. Thus, it seems that, on the one hand, some affected individuals have a strong wish to reduce further negative Covid-19 consequences and therefore want to be vaccinated to protect themselves and their surroundings [61]. On the other hand, the negative experiences that are often linked to feelings of loss of control might reduce the trust in the government and evoke reactance against further governmental measures—a well-known phenomenon in times of crisis [74, 75]. We assessed the level of being affected very generally in the present study. To better understand the two potential forms of reaction, further research should investigate which specific areas of physical and mental health as well as economic factors are affected and whether the impact is rather short-term or long-term as reactance is often evoked by long-term loss of control [75].

Considering the mental health factors, stress symptoms negatively predicted vaccination willingness in Germany and the U.S. which is in line with earlier findings on vaccination willingness [76] and on general adherence to the governmental Covid-19 measures [34, 35]. People who experience high levels of stress in extraordinary situations often tend to maladaptive reactions that can worsen the situation [77, 78]. In a recent longitudinal study from Germany, stress symptoms positively predicted a maladaptive response to the pandemic outbreak that included frustration and hopelessness [49]. Symptoms of depression and anxiety as well as psychological burden experienced by Covid-19 were not significantly associated with vaccination willingness in the nine investigated countries. This finding could reveal that negative mental health factors (exception stress symptoms) in general do not remarkably predict the willingness. However, following Bendau, Plag [15] the non-significant results could also partly be due to the fact that we used the subscales of the DASS-21 that assess general psychopathological symptoms of depression and anxiety. In contrast, studies that measured the specific Covid-19 concerns, reported significant relationships. For example specific Covid-19 related anxiety and fear of health-related consequences caused by the virus positively predicted the willingness [15, 30, 62]. Furthermore, due to the low scale reliability of the Covid-19 Burden Scale in that country, the relationships of psychological burden should be interpreted with caution in China.

PMH is a well-known protective factor that confers resilience, reduces the negative impact of depression and anxiety, and fosters self-efficacy in stressful situations [79, 80]. In line with earlier research that reported a positive association between PMH and the adherence to governmental Covid-19 measures [34], PMH positively predicted vaccination willingness in China in the present study. However, the link between PMH and willingness was negative in Germany and the U.S. Thus, it can be speculated that enhanced levels of PMH might reduce the individual Covid-19 risk perception and foster non-cooperative behavior in behavioral economics terminology [81].

Overall, our findings confirm that positive mental health and mental health problems are not just two poles of a continuum that show opposed relationships, but rather represent two distinct dimensions with specific association patterns [e.g., 82, 83]. Notably, in Germany and the U.S., the effect of stress symptoms and PMH on the willingness had the same direction.

Recent research applied the Protection Motivation Theory (PMT; [84]) to explain the importance of perceived knowledge about the Covid-19 situation for the vaccination willingness [36, 37]. According to PMT, our self-protection motivation depends on (1) threat appraisal (perceived severity of the threat; perceived personal vulnerability/risk; emotional response to the threat) and (2) coping appraisal (perceived response-efficacy—our belief about the effectiveness of the protective behavior; perceived self-efficacy—our belief about the ability to adopt the protective behavior; perceived costs of the protective behavior) [85]. The most important predictors of the motivation to adapt to the protective behavior are a high level of threat appraisal (specifically severity and vulnerability) and of efficacy (response- and self-), and a low level of perceived costs [86, 87]. Huang, Hung [36] showed that the level of perceived knowledge about Covid-19 vaccines can significantly positively foster the coping appraisal and thus indirectly the motivation for the protective behavior. Wang, Ahorsu [37] emphasized the role of the information source in this context. Specifically, the use of online sources was negatively linked to perceived knowledge, and it was positively associated with greater perceived costs. Other studies that differed between various forms of (online) sources showed that the use of official governmental sites and of traditional media such as television and newspaper reports to get Covid-19 information was positively linked to vaccination willingness [15, 88]. In contrast, the use of social media such as Facebook and Twitter contributed to less willingness [43]. Our results are in line with the available literature. Especially the use of television reports—that are available to the majority of the population in many countries and do not require specific technical or reading skills—seemed to positively contribute to the vaccination willingness in late spring 2021 (positive effect in FR, GE, RU, ES, SV, and UK). The use of newspaper reports was positively linked to willingness in Poland, and the use of official sites provided a positive effect in China and Germany. The use of social media negatively predicted vaccination willingness in Poland, Sweden, and the U.S. The main characteristics of social media are the permanent availability of unfiltered information, the freedom to create and to share online content and to engage in social interaction about this content [89]. However, these characteristics increase the risk of an uncontrollable online sharing of fake news and conspiracy believes about Covid-19 that can negatively impact the knowledge about the vaccines, increase the perceived response cost and thus reduce the motivation to adherence to governmental measures in general [90] and vaccination willingness in particular [91]. Critical concerns about potential side effects of the Covid-19 vaccines belong to the main reasons of vaccination refusal [10, 16]. These concerns can rapidly spread via social media and thus foster the negative attitude of vaccination opponents and reduce the vaccination willingness in rather ambivalent and unsure individuals [15].

The detailed consideration of the governmental Covid-19 communication and the way how people feel to be treated since the pandemic outbreak as potential willingness predictors revealed an interesting country-specific result pattern. Both seemed to be of importance especially in Poland, Sweden and the U.S. The evaluation of the governmental communication as credible and honest (SV and US) and as guided be people’s interests (PL), as well as the feeling of being well supported (PL) and of being taken seriously (US) served as positive willingness predictors. Moreover, the feeling of being left alone contributed to lower vaccination willingness in France. Overall, these findings correspond to previous research which emphasized that a positive evaluation of governmental communication among the population can foster the trust in the introduced measures and reduce worries and concerns about vaccination safety [17, 44, 92, 93]. However, we found also some rather unexpected results. Vaccination willingness was negatively predicted by the evaluation of the governmental communication as clear and understandable in Sweden and the U.S. The feeling of being well informed did not predict willingness at all. These findings might be explained by the fact that both items did not focus on the content of the communication. Presumed that the communication was informative and understandable but did not include enough arguments about the vaccination importance, a positive effect on willingness could be absent. A further unexpected result that requires replication by future research was that the feeling of being taken seriously negatively predicted vaccination willingness in Poland.

Finally, the potential predictive effect of the evaluation of the governmental Covid-19 measures as useful and the adherence to them was investigated. In line with earlier studies that reported that the believe in the effectiveness of the vaccines is a strong predictor of vaccination willingness [61, 69, 73], usefulness evaluation served as a positive predictor of willingness in all countries except China. Moreover, adherence to the measures was a positive predictor of willingness in Poland, Russia, Spain, Sweden, and the U.K.

From the present results, conclusions and recommendations can be formulated for the nine countries studied to promote vaccination willingness and thus to move closer to herd immunity. Table 5 reveals which groups should be specifically focused on by governmental programs and advertising campaigns to increase vaccination willingness. Depending on the country, female and male persons as well as younger and older people should be focused on. Also, people who have a lower social status and those who life in a large city should be focused on by the companies. It should be emphasized that the vaccination of individuals who do not belong to the Covid-19 risk group and who are not affected by the Covid-19 situation in term of physical health or mental health is also of great importance. They might underestimate the usefulness of mitigation measures including vaccination. If they also underestimate their own risk of infection, they might adhere to the measures to a lesser extent. This conclusion is in line with available literature (e.g., [29, 30]) that applied the Theory of Planned Behavior (TPB; [94]) to explain the Covid-19 vaccination willingness. According to the TPB, attitude, subjective norm, and perceived behavioral control significantly predict our behavioral intention that influences the actual behavior [94]. Recent research from China [29] and Iran [30] showed that in the Covid-19 situation especially perceived risk of infection can foster the attitude toward the vaccination willingness positively. In Taiwan, individuals who felt relatively safe tended to decline the Covid-19 vaccination [31]. Moreover, especially people who are affected economically should receive attention to reduce their feeling of being left alone by the national government and authorities and to foster their perception of vaccination and other Covid-19 mitigation measures as useful. Individuals with high stress symptoms and those with low PMH level should be focused on. Television reports, print media and official governmental sites as information source should be brought to the fore as effective Covid-19 information sources that emphasize the usefulness of the governmental measures and the urgent need of adherence to them. Moreover, Covid-19 related content provided on social media should be stronger controlled and regulated by the providers and made a topic of governmental concerns. An effort should be made to foster the credibility of the public Covid-19 communication, to stress that it is guided by people’s interest and that the population is taken seriously. This could be of specific importance for the vaccination willingness of people who lack the feeling of governmental support.

**Table 5:** Recommended focus of vaccination companies for increase of vaccination willingness based on significant effects of the logistic regression analyses.

Recommended focus of vaccination companies:	Country								
Persons who are/have/do…	CH	FR	GE	PL	RU	ES	SV	UK	US
…female	X								
…male		X	X		X				X
…younger		X							
…older	X								
…a partner	X				X	X		X	
…a lower social status		X					X		X
…habitants of large cities				X	X				
…not belong to Covid-19 risk group		X					X		X
…affected by Covid-19 in terms of physical health							X		X
…not affected by Covid-19 in terms of physical health		X			X				
…affected economically by Covid-19		X							X
…not affected mentally by Covid-19							X		
…high stress symptoms			X						X
…low level of positive mental health	X								
…high level of positive mental health			X						X
…not use television reports as Covid-19 information source		X	X		X	X	X	X	
…not use print media as Covid-19 information source				X					
…not use official sites as Covid-19 information source	X		X						
…use social media as Covid-19 information source				X			X		X
…not perceive the governmental communication as credible and honest							X		X
…not perceive the governmental communication as guided by people’s interests				X					
…feel themselves not supported by the government				X					
…feel themselves not taken seriously by the government									X
…feel themselves left alone by the government		X							
…not perceive the governmental Covid-19 measures as useful		X	X	X	X	X	X	X	X
…not adhere to the governmental Covid-19 measures				X	X	X	X	X	

CH = China, FR = France, GE = Germany, PL = Poland, RU = Russia, ES = Spain, SV = Sweden, UK = the U.K., US = the U.S.

While our study has several strengths (large representative samples on three continents, measures with established reliability, timeliness of the investigated issues), it also has limitations that need to be taken into account. First, all data were assessed at the same measurement time point. The cross-sectional design of the present study does not allow true conclusions on causality. Therefore, the effectiveness of our country-specific suggestions for the improvement of the vaccination willingness should be investigated by future experimental research. Second, the representativeness of our findings could be enhanced by the inclusion of further variables for the stratification such as level of education, income, and marital status. Third, the present results represent a snapshot of the Covid-19 situation in the late spring of 2021 only in nine mostly European countries while for example African or South American countries were not included. This limits the generalizability of our results. Moreover, even though the present data were assessed by YouGov who implemented age, gender, and region stratification. In China, the representativeness of age was not achieved. Younger people are remarkably over-represented (see demographic data, Table 1). Fourth, overall, the investigated predictors of vaccination willingness showed the lowest effects in China. This can partly be explained by the smaller number of assessed predictors and the low reliability of the burden measure. However, it can also be that—due to sociocultural circumstances—further factors that were not included in the present study such as for example personality traits (e.g., the Big Five; [95]) are more important for the vaccination willingness in China than in the other eight countries. Fifth, we can only speculate about the reasons for the vaccination willingness or the vaccination refusal because we did not assess them directly. Sixth, we assessed Covid-19 vaccination willingness with only one item that we formulated for the present study. As shown by previous research, the formulation of the question about the willingness can significantly influence the response and it can result in an underestimation of the rate of the vaccination willingness [14]. Furthermore, even though single-item scales are time and cost efficient instruments [96, 97], future studies should replicate our findings using longer multidimensional measures for the assessment of vaccination willingness. For example, they could use the new developed and validated Drivers of Covid-19 Vaccination Acceptance Scale (DrVac-COVID19S) that consists of four main factors (values, impacts, knowledge, and autonomy) and twelve items [98, 99]. A replication of the current finding with this instrument would contribute to the validation of our single-item measure.

In summary, the current cross-national study shows that in general the Covid-19 vaccination willingness across various countries was about 80% in spring 2021. The lowest willingness was in Russia where the overall adherence to governmental Covid-19 measures was already lower than in the other included countries in summer 2020 before the availability of a vaccine. The highest vaccination willingness was in the U.K.–the country with the highest adherence to governmental measures in summer 2020 [34].

Willingness is predicted by various factors, such as age, gender, living environment, mental health variables, the quality of government Covid-19 communication, the source of the Covid-19 information, the evaluation of the governmental measures as useful and the adherence to them. The patterns of prediction, however, are rather country-specific. To increase vaccination willingness and thus to reach a global heard immunity against Covid-19, each government must address the specific pattern for its population. This is the main way to contribute to the success of the “Project Lightspeed” and further projects that worked and are working on the development of Covid-19 vaccines around the globe.

## Supporting information

S1 Dataset Dataset used for analyses in present study. (SAV)

## References

[1] BioNTech. Aiming to address the global corinavirus pandemic: Project Lightspeed 2021. https://biontech.de/covid-19-portal/project-lightspeed.

[2] Lamb YN. BNT162b2 mRNA COVID-19 vaccine: First approval. Drugs. 2021;81: 495–501. doi: 10.1007/s40265-021-01480-7 33683637 PMC7938284

[3] Garland M. How Pfizer transformed its supply chain to deliver vaccines at lightspeed. 2021. https://www.supplychaindive.com/news/pfizer-jim-cafone-covid-vaccine-manufacturing-distribution-cscmp/606867/

[4] World Health Organization. Status of COVID-19 Vaccines 2021. https://www.who.int/emergencies/diseases/novel-coronavirus-2019/covid-19-vaccines.

[5] Basta NE, Moodie EEM. Vaccines Candidates by Trial Phase 2021. https://covid19.trackvaccines.org/vaccines/.

[6] Alimoradi Z, Lin C-Y, Pakpour AH. Coronavirus disease-19 vaccine inequity and gross domestic product. Asian Journal of Social Health and Behavior. 2021;4(4): 129–30.

[7] Randolph HE, Barreiro LB. Herd immunity: understanding COVID-19. Immunity. 2020;52(5): 737–41. doi: 10.1016/j.immuni.2020.04.012 32433946 PMC7236739

[8] Neumann-Böhme S, Varghese NE, Sabat I, Barros PP, Brouwer W, van Exel J, et al. Once we have it, will we use it? A European survey on willingness to be vaccinated against covid-19. The European Journal of Health Economics. 2020;21(7): 977–82. doi: 10.1007/s10198-020-01208-6 32591957 PMC7317261

[9] Lazarus JV, Ratzan SC, Palayew A, Gostin LO, Larson HJ, Rabin K, et al. A global survey of potential acceptance of a COVID-19 vaccine. Nature Medicine. 2020: 1–4. 10.1038/s41591-020-1124-9 PMC7573523 33082575

[10] Chen M, Li Y, Chen J, Wen Z, Feng F, Zou H, et al. An online survey of the attitude and willingness of Chinese adults to receive COVID-19 vaccination. Human Vaccines & Immunotherapeutics. 2021;17(7): 2279–88. doi: 10.1080/21645515.2020.1853449 33522405 PMC8189089

[11] Liu R, Zhang Y, Nicholas S, Leng A, Maitland E, Wang J. COVID-19 Vaccination Willingness among Chinese Adults under the Free Vaccination Policy. Vaccines. 2021;9(3): 292–301. doi: 10.3390/vaccines9030292 33801136 PMC8004171

[12] Kelly BJ, Southwell BG, McCormack LA, Bann CM, MacDonald PDM, Frasier AM, et al. Predictors of willingness to get a COVID-19 vaccine in the US. BMC Infectious Diseases. 2021;21(1): 1–7. 33845781 10.1186/s12879-021-06023-9 PMC8039496

[13] Sherman SM, Smith LE, Sim J, Amlôt R, Cutts M, Dasch H, et al. COVID-19 vaccination intention in the UK: results from the COVID-19 vaccination acceptability study (CoVAccS), a nationally representative cross-sectional survey. Human Vaccines & Immunotherapeutics. 2021;17(6): 1612–21. doi: 10.1080/21645515.2020.1846397 33242386 PMC8115754

[14] Rieger MO. Willingness to vaccinate against COVID-19 might be systematically underestimated. Asian Journal of Social Health and Behavior. 2021;4(2): 81–3. doi: 10.4103/shb.shb_7_21

[15] Bendau A, Plag J, Petzold MB, Ströhle A. COVID-19 vaccine hesitancy and related fears and anxiety. International Immunopharmacology. 2021;97: 107724. 33951558 10.1016/j.intimp.2021.107724 PMC8078903

[16] Daly M, Robinson E. Willingness to vaccinate against COVID-19 in the US: representative longitudinal evidence from April to October 2020. American Journal of Preventive Medicine. 2021;60(6): 766–73. 33773862 10.1016/j.amepre.2021.01.008 PMC7883746

[17] Tran VD, Pak TV, Gribkova EI, Galkina GA, Loskutova EE, Dorofeeva VV, et al. Determinants of COVID-19 vaccine acceptance in a high infection-rate country: a cross-sectional study in Russia. Pharmacy Practice (Granada). 2021;19(1): 2276–85. doi: 10.18549/pharmpract.2021.1.2276 33828622 PMC8005327

[18] Rodríguez-Blanco N, Montero-Navarro S, Botella-Rico JM, Felipe-Gómez AJ, Sánchez-Más J, Tuells J. Willingness to Be Vaccinated against COVID-19 in Spain before the Start of Vaccination: A Cross-Sectional Study. International Journal of Environmental Research and Public Health. 2021;18(10): 5272–87. doi: 10.3390/ijerph18105272 34063476 PMC8155897

[19] Lazarus JV, Wyka K, Rauh L, Rabin K, Ratzan S, Gostin LO, et al. Hesitant or not? The association of age, gender, and education with potential acceptance of a COVID-19 vaccine: A country-level analysis. Journal of Health Communication. 2020;25(10): 799–807. doi: 10.1080/10810730.2020.1868630 33719881

[20] Feleszko W, Lewulis P, Czarnecki A, Waszkiewicz P. Flattening the curve of covid-19 vaccine rejection—An international overview. Vaccines. 2021;9(1): 44–51. doi: 10.3390/vaccines9010044 33451104 PMC7828585

[21] Scholten S, Velten J, Margraf J. Mental distress and perceived wealth, justice and freedom across eight countries: The invisible power of the macrosystem. PloS One. 2018;13(5): e0194642. doi: 10.1371/journal.pone.0194642 29718911 PMC5931469

[22] Scholten S, Velten J, Neher T, Margraf J. Wealth, justice and freedom: objective and subjective measures predicting poor mental health in a study across eight countries. SSM-Population Health. 2017;3: 639–48. doi: 10.1016/j.ssmph.2017.07.010 29349252 PMC5769050

[23] Margraf J, Lavallee KL, Zhang XC, Woike JK, Schneider S. Mental health and the wish to have a child: a longitudinal, cross-cultural comparison between Germany and China. Journal of Psychosomatic Obstetrics & Gynecology. 2020: 1–13. doi: 10.1080/0167482X.2020.1816959 32914664

[24] Margraf J, Brailovskaia J, Schneider S. Adherence to behavioral Covid-19 mitigation measures strongly predicts mortality. Plos One. 2021;16(3): e0249392. doi: 10.1371/journal.pone.0249392 33784361 PMC8009358

[25] Gandhi M, Rutherford GW. Facial Masking for Covid-19—Potential for “Variolation” as We Await a Vaccine. New England Journal of Medicine. 2020;383(18): e101. doi: 10.1056/NEJMp2026913 32897661 PMC7890559

[26] Tso RV, Cowling BJ. Importance of face masks for COVID-19–a call for effective public education. Clinical Infectious Diseases. 2020;71(16): 2195–8. doi: 10.1093/cid/ciaa593 32614045 PMC7337661

[27] Kumari A, Ranjan P, Chopra S, Kaur D, Kaur T, Upadhyay AD, et al. Knowledge, barriers and facilitators regarding COVID-19 vaccine and vaccination programme among the general population: A cross-sectional survey from one thousand two hundred and forty-nine participants. Diabetes & Metabolic Syndrome: Clinical Research & Reviews. 2021;15(3): 987–92. doi: 10.1016/j.dsx.2021.04.015 33984818 PMC8087578

[28] Yoda T, Katsuyama H. Willingness to receive covid-19 vaccination in Japan. Vaccines. 2021;9(1): 48–55. doi: 10.3390/vaccines9010048 33466675 PMC7828811

[29] Fan C-W, Chen IH, Ko N-Y, Yen C-F, Lin C-Y, Griffiths MD, et al. Extended theory of planned behavior in explaining the intention to COVID-19 vaccination uptake among mainland Chinese university students: An online survey study. Human Vaccines & Immunotherapeutics. 2021;17(10): 3413–20. doi: 10.1080/21645515.2021.1933687 34170792 PMC8437493

[30] Yahaghi R, Ahmadizade S, Fotuhi R, Taherkhani E, Ranjbaran M, Buchali Z, et al. Fear of COVID-19 and Perceived COVID-19 Infectability Supplement Theory of Planned Behavior to Explain Iranians’ Intention to Get COVID-19 Vaccinated. Vaccines. 2021;9(7): 684–98. doi: 10.3390/vaccines9070684 34206226 PMC8310138

[31] Kukreti S, Lu M-Y, Lin Y-H, Strong C, Lin C-Y, Ko N-Y, et al. Willingness of Taiwan’s healthcare workers and outpatients to vaccinate against COVID-19 during a period without community outbreaks. Vaccines. 2021;9(3): 246–55. doi: 10.3390/vaccines9030246 33808950 PMC8000386

[32] Nohl A, Afflerbach C, Lurz C, Brune B, Ohmann T, Weichert V, et al. Acceptance of COVID-19 Vaccination among Front-Line Health Care Workers: A Nationwide Survey of Emergency Medical Services Personnel from Germany. Vaccines. 2021;9(5): 424–235. doi: 10.3390/vaccines9050424 33922812 PMC8144974

[33] Lukat J, Margraf J, Lutz R, van der Veld WM, Becker ES. Psychometric properties of the positive mental health scale (PMH-scale). BMC Psychology. 2016;4(1): 8. doi: 10.1186/s40359-016-0111-x 26865173 PMC4748628

[34] Margraf J, Brailovskaia J, Schneider S. Behavioral measures to fight COVID-19: An 8-country study of perceived usefulness, adherence and their predictors. PLoS One. 2020;15(12): e0243523. doi: 10.1371/journal.pone.0243523 33284865 PMC7721173

[35] Pollak Y, Dayan H, Shoham R, Berger I. Predictors of adherence to public health instructions during the COVID-19 pandemic. Psychiatry and Clinical Neurosciences. 2020;74(11): 602–4. doi: 10.1111/pcn.13122 32729646

[36] Huang P-C, Hung C-H, Kuo Y-J, Chen Y-P, Ahorsu DK, Yen C-F, et al. Expanding protection motivation theory to explain willingness of COVID-19 vaccination uptake among Taiwanese university students. Vaccines. 2021;9(9): 1046–59. doi: 10.3390/vaccines9091046 34579283 PMC8473221

[37] Wang P-W, Ahorsu DK, Lin C-Y, Chen IH, Yen C-F, Kuo Y-J, et al. Motivation to have covid-19 vaccination explained using an extended protection motivation theory among university students in china: The role of information sources. Vaccines. 2021;9(4): 380–94. doi: 10.3390/vaccines9040380 33924604 PMC8070343

[38] Whiting A, Williams D. Why people use social media: a uses and gratifications approach. Qualitative Market Research: An International Journal. 2013;16(4): 362–9. doi: 10.1108/QMR-06-2013-0041

[39] Kouzy R, Abi Jaoude J, Kraitem A, El Alam MB, Karam B, Adib E, et al. Coronavirus goes viral: quantifying the COVID-19 misinformation epidemic on Twitter. Cureus. 2020;12(3): e7255. doi: 10.7759/cureus.7255 32292669 PMC7152572

[40] Apuke OD, Omar B. Fake news and COVID-19: modelling the predictors of fake news sharing among social media users. Telematics and Informatics. 2021;56: 101475. doi: 10.1016/j.tele.2020.101475 PMC7390799 34887612

[41] Budhwani H, Sun R. Creating COVID-19 stigma by referencing the novel coronavirus as the “Chinese virus” on Twitter: quantitative analysis of social media data. Journal of Medical Internet Research. 2020;22(5): e19301. 32343669 10.2196/19301 PMC7205030

[42] Depoux A, Martin S, Karafillakis E, Bsd RP, Wilder-Smith A, Larson H. The pandemic of social media panic travels faster than the COVID-19 outbreak. Journal of Travel Medicine. 2020;27(3): 1–2. doi: 10.1093/jtm/taaa031 32125413 PMC7107516

[43] Jennings W, Stoker G, Willis H, Valgardsson V, Gaskell J, Devine D, et al. Lack of trust and social media echo chambers predict COVID-19 vaccine hesitancy. Vaccines. 2021;9(6): 593–616. doi: 10.3390/vaccines9060593 34204971 PMC8226842

[44] Chew NWS, Cheong C, Kong G, Phua K, Ngiam JN, Tan BYQ, et al. An Asia-Pacific study on healthcare workers’ perceptions of, and willingness to receive, the COVID-19 vaccination. International Journal of Infectious Diseases. 2021;106: 52–60. doi: 10.1016/j.ijid.2021.03.069 33781902 PMC7997703

[45] Mayr S, Erdfelder E, Buchner A, Faul F. A short tutorial of GPower. Tutorials in Quantitative Methods for Psychology. 2007;3(2): 51–9. doi: 10.20982/tqmp.03.2.p051

[46] Velten J, Scholten S, Brailovskaia J, Margraf J. Psychometric properties of the S-Scale: Assessing a psychological mindset that mediates the relationship between socioeconomic status and depression. PLoS One. 2021;16(10): e0258333. doi: 10.1371/journal.pone.0258333 34648554 PMC8516301

[47] Brailovskaia J, Cosci F, Mansueto G, Margraf J. The relationship between social media use, stress symptoms and burden caused by Coronavirus (Covid-19) in Germany and Italy: A cross-sectional and longitudinal investigation. Journal of Affective Disorders Reports. 2021;3: 100067. doi: 10.1016/j.jadr.2020.100067 PMC8995101 35434690

[48] Lovibond PF, Lovibond SH. The structure of negative emotional states: comparison of the Depression Anxiety Stress Scales (DASS) with the Beck Depression and Anxiety Inventories. Behaviour Research and Therapy. 1995;33(3): 335–43. doi: 10.1016/0005-7967(94)00075-u 7726811

[49] Brailovskaia J, Margraf J. Predicting adaptive and maladaptive responses to the Coronavirus (COVID-19) outbreak: A prospective longitudinal study. International Journal of Clinical and Health Psychology. 2020;20(3): 181–91. doi: 10.1016/j.ijchp.2020.06.002 32837518 PMC7321043

[50] Bieda A, Hirschfeld G, Schönfeld P, Brailovskaia J, Zhang XC, Margraf J. Universal Happiness? Cross-Cultural Measurement Invariance of Scales Assessing Positive Mental Health. Psychological Assessment. 2017;29(4): 408–21. doi: 10.1037/pas0000353 27322203

[51] Cai D, Zhu M, Lin M, Zhang XC, Margraf J. The bidirectional relationship between positive mental health and social rhythm in college students: a three-year longitudinal study. Frontiers in Psychology. 2017;8: 1–7. 28713318 10.3389/fpsyg.2017.01119 PMC5492866

[52] Scholten S, Velten J, Bieda A, Zhang XC, Margraf J. Testing measurement invariance of the Depression, Anxiety, and Stress Scales (DASS-21) across four countries. Psychological Assessment. 2017;29(11): 1376–90. doi: 10.1037/pas0000440 28125249

[53] Berry JW. Introduction to methodology. In: Triandis H, Berry JW, editors. Handbook of cross-cultural psychology 2. Boston: Allyn & Bacon; 1989. p. 1–28

[54] IBM Corp. IBM SPSS Statistics for Windows, Version 26.0. Armonk, NY: IBM Corp; 2019.

[55] Cohen J. Statistical power analysis for the behavioral sciences. 2nd ed. Hillsdale, NJ: Lawrence Erlbaum; 1988.

[56] Worldometers. Countries in the world by population. 2021. https://www.worldometers.info/world-population/population-by-country/

[57] Verger P, Scronias D, Dauby N, Adedzi KA, Gobert C, Bergeat M, et al. Attitudes of healthcare workers towards COVID-19 vaccination: a survey in France and French-speaking parts of Belgium and Canada, 2020. Eurosurveillance. 2021;26(3): 1–8. doi: 10.2807/1560-7917.ES.2021.26.3.2002047 33478623 PMC7848677

[58] Dodd RH, Cvejic E, Bonner C, Pickles K, McCaffery KJ, Ayre J, et al. Willingness to vaccinate against COVID-19 in Australia. The Lancet Infectious Diseases. 2021;21(3): 318–9. doi: 10.1016/S1473-3099(20)30559-4 32619436 PMC7326391

[59] Griner J, Sobol A. Chinese Students’ Motivations for Studying Abroad. Global Studies Journal. 2014;7(1): 2–14.

[60] Henze J, Zhu J. Current research on Chinese students studying abroad. Research in Comparative and International Education. 2012;7(1): 90–104. doi: 10.2304/rcie.2012.7.1.90

[61] Machida M, Nakamura I, Kojima T, Saito R, Nakaya T, Hanibuchi T, et al. Acceptance of a covid-19 vaccine in Japan during the covid-19 pandemic. Vaccines. 2021;9(3): 210–20. doi: 10.3390/vaccines9030210 33802285 PMC8002097

[62] Detoc M, Bruel S, Frappe P, Tardy B, Botelho-Nevers E, Gagneux-Brunon A. Intention to participate in a COVID-19 vaccine clinical trial and to get vaccinated against COVID-19 in France during the pandemic. Vaccine. 2020;38(45): 7002–6. doi: 10.1016/j.vaccine.2020.09.041 32988688 PMC7498238

[63] Mills MC, Salisbury D. The challenges of distributing COVID-19 vaccinations. EClinicalMedicine. 2021;31: 100674. doi: 10.1016/j.eclinm.2020.100674 33319186 PMC7725651

[64] Zuckerman M. Sensation Seeking (Psychology Revivals): Beyond the Optimal Level of Arousal. New York, NY: Psychology Press; 2014.

[65] Romero-Rodríguez J-M, Aznar-Díaz I, Marín-Marín J-A, Soler-Costa R, Rodríguez-Jiménez C. Impact of problematic smartphone use and Instagram use intensity on self-esteem with university students from physical education. International Journal of Environmental Research and Public Health. 2020;17(12): 4336–45. doi: 10.3390/ijerph17124336 32560447 PMC7344735

[66] Ryan KM, Weikel K, Sprechini G. Gender differences in narcissism and courtship violence in dating couples. Sex Roles. 2008;58(11–12): 802–13. doi: 10.1007/s11199-008-9403-9

[67] Zeballos Rivas DR, Lopez Jaldin ML, Nina Canaviri B, Portugal Escalante LF, Alanes Fernández AMC, Aguilar Ticona JP. Social media exposure, risk perception, preventive behaviors and attitudes during the COVID-19 epidemic in La Paz, Bolivia: A cross sectional study. PloS One. 2021;16(1): e0245859. doi: 10.1371/journal.pone.0245859 33481945 PMC7822287

[68] Dzieciolowska S, Hamel D, Gadio S, Dionne M, Gagnon D, Robitaille L, et al. Covid-19 vaccine acceptance, hesitancy, and refusal among Canadian healthcare workers: A multicenter survey. American Journal of Infection Control. 2021. doi: 10.1016/j.ajic.2021.04.079 33930516 PMC8079260

[69] Wang J, Jing R, Lai X, Zhang H, Lyu Y, Knoll MD, et al. Acceptance of COVID-19 Vaccination during the COVID-19 Pandemic in China. Vaccines. 2020;8(3): 482–95. doi: 10.3390/vaccines8030482 32867224 PMC7565574

[70] Popovich L, Potapchik E, Shishkin S, Richardson E, Vacroux A, Mathivet B. Russian Federation: health system review. Copenhagen, Denmark: World Health Organization. Regional Office for Europe; 2011. 22455875

[71] Li X, Krumholz HM, Yip W, Cheng KK, De Maeseneer J, Meng Q, et al. Quality of primary health care in China: challenges and recommendations. The Lancet. 2020;395(10239): 1802–12. doi: 10.1016/S0140-6736(20)30122-7 32505251 PMC7272159

[72] Shishkin SV. Russian health care system: Reforms or crisis? Journal of the New Economic Association. 2014;23(3): 162–4.

[73] Reiter PL, Pennell ML, Katz ML. Acceptability of a COVID-19 vaccine among adults in the United States: How many people would get vaccinated? Vaccine. 2020;38(42): 6500–7. doi: 10.1016/j.vaccine.2020.08.043 32863069 PMC7440153

[74] Wortman CB, Brehm JW. Responses to uncontrollable outcomes: An integration of reactance theory and the learned helplessness model. In: Berkowitz L, editor. Advances in Experimental Pocial Psychology. 8. New York: Elsevier; 1975. p. 277–336

[75] Mühlberger C, Jonas E. Reactance theory. In: Sassenberg K, Vliek MLW, editors. Social Psychology in Action. Switzerland: Springer; 2019. p. 79–94

[76] Xu Z, Shi L, Wang Y, Zhang J, Huang L, Zhang C, et al. Pathological findings of COVID-19 associated with acute respiratory distress syndrome. The Lancet Respiratory Medicine. 2020;8(4): 420–2. doi: 10.1016/S2213-2600(20)30076-X 32085846 PMC7164771

[77] Parrish BP, Cohen LH, Laurenceau J-P. Prospective relationship between negative affective reactivity to daily stress and depressive symptoms. Journal of Social and Clinical Psychology. 2011;30(3): 270–96. doi: 10.1521/jscp.2011.30.3.270

[78] Seligman MEP. Learned helplessness. Annual Review of Medicine. 1972;23(1): 407–12. doi: 10.1146/annurev.me.23.020172.002203 4566487

[79] Truskauskaite-Kuneviciene I, Kazlauskas E, Ostreikaite-Jurevice R, Brailovskaia J, Margraf J. Positive mental health and adjustment following life-stressors among young adults. Current Psychology. 2020. doi: 10.1007/s12144-020-00714-3

[80] Teismann T, Brailovskaia J, Totzeck C, Wannemüller A, Margraf J. Predictors of remission from panic disorder, agoraphobia and specific phobia in outpatients receiving exposure therapy: The importance of positive mental health. Behaviour Research and Therapy. 2018;108: 40–4. doi: 10.1016/j.brat.2018.06.006 29981937

[81] Benchekroun H, Van Long N. The build-up of cooperative behavior among non-cooperative selfish agents. Journal of Economic Behavior & Organization. 2008;67(1): 239–52. doi: 10.1016/j.jebo.2006.08.010

[82] Suldo SM, Shaffer EJ. Looking beyond psychopathology: The dual-factor model of mental health in youth. School Psychology Review. 2008;37(1): 52–68. doi: 10.1080/02796015.2008.12087908

[83] Keyes CL. Mental illness and/or mental health? Investigating axioms of the complete state model of health. Journal of Consulting and Clinical Psychology. 2005;73(3): 539–48. doi: 10.1037/0022-006X.73.3.539 15982151

[84] Rogers RW. Cognitive and physiological processes in fear appeals and attitude change: A revised theory of protection motivation. In: Cacioppo J, Petty R, editors. Social Psychophysiology. New York, NY: Guilford Press; 1983. p. 153–76

[85] Floyd DL, Prentice-Dunn S, Rogers RW. A meta-analysis of research on protection motivation theory. Journal of Applied Social Psychology. 2000;30(2): 407–29. doi: 10.1111/j.1559-1816.2000.tb02323.x

[86] Milne S, Sheeran P, Orbell S. Prediction and intervention in health-related behavior: A meta-analytic review of protection motivation theory. Journal of Applied Social Psychology. 2000;30(1): 106–43. doi: 10.1111/j.1559-1816.2000.tb02308.x

[87] Teasdale E, Yardley L, Schlotz W, Michie S. The importance of coping appraisal in behavioural responses to pandemic flu. British Journal of Health Psychology. 2012;17(1): 44–59. doi: 10.1111/j.2044-8287.2011.02017.x 22233104

[88] Alley SJ, Stanton R, Browne M, To QG, Khalesi S, Williams SL, et al. As the pandemic progresses, how does willingness to vaccinate against COVID-19 evolve? International Journal of Environmental Research and Public Health. 2021;18(2): 797. doi: 10.3390/ijerph18020797 33477825 PMC7832839

[89] Oberst U, Wegmann E, Stodt B, Brand M, Chamarro A. Negative consequences from heavy social networking in adolescents: The mediating role of fear of missing out. Journal of Adolescence. 2017;55: 51–60. doi: 10.1016/j.adolescence.2016.12.008 28033503

[90] Pennycook G, McPhetres J, Zhang Y, Lu JG, Rand DG. Fighting COVID-19 misinformation on social media: Experimental evidence for a scalable accuracy-nudge intervention. Psychological Science. 2020;31(7): 770–80. doi: 10.1177/0956797620939054 32603243 PMC7366427

[91] Jennings W, Stoker G, Bunting H, Valgarðsson VO, Gaskell J, Devine D, et al. Lack of Trust, Conspiracy Beliefs, and Social Media Use Predict COVID-19 Vaccine Hesitancy. Vaccines. 2021;9(6): 593. doi: 10.3390/vaccines9060593 34204971 PMC8226842

[92] Allington D, Duffy B, Wessely S, Dhavan N, Rubin J. Health-protective behaviour, social media usage and conspiracy belief during the COVID-19 public health emergency. Psychological Medicine. 2020: 1–7. doi: 10.1017/S003329172000224X 32513320 PMC7298098

[93] Schernhammer E, Weitzer J, Laubichler MD, Birmann BM, Bertau M, Zenk L, et al. Correlates of COVID-19 vaccine hesitancy in Austria: trust and the government. Journal of Public Health 2021;42(4): 688–95. doi: 10.1093/pubmed/fdab122 33948665 PMC8135852

[94] Ajzen I. The theory of planned behavior. Organizational Behavior and Human Decision Processes. 1991;50: 179–211.

[95] Wang J-L, Jackson LA, Zhang D-J, Su Z-Q. The relationships among the Big Five Personality factors, self-esteem, narcissism, and sensation-seeking to Chinese University students’ uses of social networking sites (SNSs). Comput Human Behav. 2012;28(6): 2313–9.

[96] Brailovskaia J, Margraf J. How to measure self-esteem with one item? Validation of the German single-item self-esteem scale (G-SISE). Current Psychology. 2020;39(6): 2192–202. doi: 10.1007/s12144-018-9911-x

[97] Milton K, Bull FC, Bauman A. Reliability and validity testing of a single-item physical activity measure. British Journal of Sports Medicine. 2011;45(3): 203–8. doi: 10.1136/bjsm.2009.068395 20484314

[98] Chen IH, Ahorsu DK, Ko N-Y, Yen C-F, Lin C-Y, Griffiths MD, et al. Adapting the Motors of Influenza Vaccination Acceptance Scale into the Motors of COVID-19 Vaccination Acceptance Scale: Psychometric evaluation among mainland Chinese university students. Vaccine. 2021;39: 4510–5. doi: 10.1016/j.vaccine.2021.06.044 34217571 PMC8216877

[99] Yeh Y-C, Chen IH, Ahorsu DK, Ko N-Y, Chen K-L, Li P-C, et al. Measurement invariance of the drivers of covid-19 vaccination acceptance scale: Comparison between taiwanese and mainland chinese-speaking populations. Vaccines. 2021;9(3): 297–315. doi: 10.3390/vaccines9030297 33810036 PMC8004810

