# Supplementary material for: To vaccinate or not to vaccinate!? Predictors of willingness to receive Covid-19 vaccination in Europe, the U.S., and China
Source: PLoS One. 2021 Dec 1;16(12):e0260230. doi: 10.1371/journal.pone.0260230 (PMC8635370; doi:10.1371/journal.pone.0260230)
Supplement: Attachment — Submitted filename: Review_PONE-D-21-31209.docx [file pone.0260230.s002.docx]

**Reviewer#**

**Comments to the Author**

**Summary**

The study aimed to investigate the vaccination willingness and its potential predictors in representative online samples in nine countries (China, France, Germany, Poland, Russia, Spain, Sweden, U.K., U.S.). The overall willingness among the general population was 79%. The highest willingness was in the U.K., followed by Spain and China, the lowest in Russia. Further, factors like demographic variables, mental and physical health status, evaluation of governmental communication, social media use, and general adherence to Covid-19 measures showed a country-specific predictive pattern.

However, there still were some notable deficiencies in the article.

**Introduction**

1. Page no.3, Line no. 68: It is better to use *general population* willingness instead of people’s willingness.
2. The structure of the introduction section is very confusing; the author has first mentioned aim, then kept explaining the literature gap, and then again started talking about aim two. I would suggest restructuring the introduction for better understanding.

**Methods and results**

3. It is not clear how the author has categorized social status? what is working class? How is it different from other classes in terms of status?
4. How have the authors validated the COVID-specific questionnaire?
5. What is the response rate in the study?
6. What were all languages used during questionnaire administration? The questionnaire used in the study was in English, or was it translated?
7. Please specify n.s in table number 3

**Discussion**

8. What will be the possible reason for the lower willingness level among China in this study? Line 389-391, page no.19
9. What does the author mean by physical intervention here? Line411, page no.20
10. What does the author mean by correlative design here?
