# Supplementary material for: To vaccinate or not to vaccinate!? Predictors of willingness to receive Covid-19 vaccination in Europe, the U.S., and China
Source: PLoS One. 2021 Dec 1;16(12):e0260230. doi: 10.1371/journal.pone.0260230 (PMC8635370; doi:10.1371/journal.pone.0260230)
Supplement: Attachment — Submitted filename: Response to Reviewers.docx [file pone.0260230.s003.docx]

List of changes:

| Comment of the Editor/Reviewer | Change – page references refer to the revised manuscript | Our Comment |
| --- | --- | --- |
| *Editor* |  |  |
| The cross-country comparisons of the vaccination uptake willingness is important as we know that the herd immunity should be better to achieve around the world. Then, we can have more country interaction and reactivate the economic communications. Therefore, I believe that the present study tackle an important and timely topic. An expert in this area has reviewed this work and provides good comments for you to work on. In general, I agree with all of her opinions and hope that all these comments can help you improve the presentation. | Please, see answers to the reviewers. | Thank you very much for the possibility to submit a revised version of our manuscript. We have considerably revised the original manuscript by addressing each point raised by you and the reviewer, which improved the quality of our manuscript considerably. We really hope to have covered all the suggestions adequately. |
| I would especially follow her suggestion regarding the Introduction structure. Indeed, please put all the aims, purposes, and hypotheses toward the end of the Introduction. In this way, the readability can be improved. | See for example:  *Page 3:*  “The success of the vaccination campaigns depends on the availability of the vaccines and their allocation that is positively associated with the income of a specific country [6].”  *Page 4:*  “In Germany, a willingness of 83.6% was assessed in December 2020 [14] and of 78.3% in January 2021 [15].”  *Page 5:*  “Second, belonging to the Covid-19 risk group (e.g., pre-existing health conditions, age-related, weakened immune system), risk perception and perceived Covid-19 infectability served as positive willingness predictors in China [29], Iran [30], Spain [18] and Taiwan [31].”  *Page 6:*  “In Germany and Iran, the psychological burden caused by the Covid-19 situation and the anxiety of health-related consequences caused by the virus were identified as its positive predictors [15, 30, 32].”  *Page 6:*  “Third, available research shows that being well-informed about the vaccines, herd immunity and further factors that are linked to the pandemic is an important predictor of the Covid-19 vaccination willingness [14, 36, 37].” | Considering your concern and the concern of the reviewer, we considerably overworked the structure of the Introduction section in the revised manuscript. Thank you very much for the suggested references. We have incorporated the different references in the overworked Introduction section. They strengthened our work. Now, we first describe the importance of the Covid-19 vaccination willingness and the literature gap; then, we present the two main aims of our study – 1. To assess the willingness in the nine countries simultaneously and to compare it between the countries, 2. To investigate potential predictors of the willingness; then, we explain the potential predictors that we included in our investigation. We hope that the Introduction section is clearer now. |
| Moreover, I would like the authors to mention the following issues in the Introduction. First, please mention that there is some debate regarding the willingness of COVID-19 vaccination uptake with relevant ciations (e.g., Kukreti et al. found some low levels of willingness in some populations, while Rieger argues that willingness of COVID-19 vaccination uptake might be underestimated). Second, please mention that there may have inequity issue for the vaccination.  References:  Kukreti, S., Lu, M.-Y., Lin, Y.-H., Strong, C., Lin, C.-Y., Ko, N.-Y., Chen, P.-L., & Ko, W.-C. (2021). Willingness of Taiwan’s healthcare workers and outpatients to vaccinate against COVID-19 during a period without community outbreaks. Vaccines, 9(3), 246.  Rieger MO. Willingness to vaccinate against COVID-19 might be systematically underestimated. Asian J Soc Health Behav 2021;4:81-3.  Alimoradi Z, Lin CY, Pakpour AH. Coronavirus disease-19 vaccine inequity and gross domestic product. Asian J Soc Health Behav 2021;4:129-30 | *Page 3:*  “The success of the vaccination campaigns depends on the availability of the vaccines and their allocation that is positively associated with the income of a specific country [6].”  *Page 4:*  “In Germany, a willingness of 83.6% was assessed in December 2020 [14] and of 78.3% in January 2021 [15].”  *Page 5:*  “Second, belonging to the Covid-19 risk group (e.g., pre-existing health conditions, age-related, weakened immune system), risk perception and perceived Covid-19 infectability served as positive willingness predictors in China [29], Iran [30], Spain [18] and Taiwan [31].”  *Page 6:*  “Third, available research shows that being well-informed about the vaccines, herd immunity and further factors that are linked to the pandemic is an important predictor of the Covid-19 vaccination willingness [14, 36, 37].”  *Page 29:*  “In Taiwan, individuals who felt relatively safe tended to decline the Covid-19 vaccination [31].”  *Page 32:*  “Sixth, we assessed Covid-19 vaccination willingness with only one item that we formulated for the present study. As shown by previous research, the formulation of the question about the willingness can significantly influence the response and it can result in an underestimation of the rate of the vaccination willingness [14].” | Following your advice and the advice of the reviewer, we considerably overworked the structure of the Introduction section in the revised manuscript. Thank you very much for the suggestion of the three references. We included all of them in the overworked Introduction section. Moreover, we included them also in the Discussion section. |
| Apart from the Introduction, please mention in the Discussion regarding the following two points.  First, there are existing behavior theories that have been tested to understand the vaccination uptake. The authors should discuss the possibilities regarding how to incorporate their predictor findings to these theories to maximize the possibilities in increasing people's willingness to uptake COVID-19 vaccine. Please see the following references:  Fan, C.-W., Chen, I.-H., Ko, N.-Y., Yen, C.-F., Lin, C.-Y., Griffiths, M. D., & Pakpour, A. H. (2021).  Extended theory of planned behavior in explaining the intention to COVID-19 vaccination uptake among mainland Chinese university students: An online survey study. Human Vaccines & Immunotherapeutics, 17(10), 3413-3420.  Yahaghi, R., Ahmadizade, S., Fotuhi, R., Taherkhani, E., Ranjbaran, M., Buchali, Z., Jafari, R., Zamani, N., Shahbazkhania, A., Simiari, H., Rahmani, J., Yazdi, N., Alijani, H., Poorzolfaghar, L., Rajabi, F., Lin, C.-Y., Broström, A., Griffiths, M. D., Pakpour, A. H. (2021). Fear of COVID-19 and perceived COVID-19 infectability supplement Theory of Planned Behavior to explain Iranians’ intention to get COVID-19 vaccinated. Vaccines, 9, 684.  Huang, P.-C., Hung, C.-H., Kuo, Y.-J., Chen, Y.-P., Ahorsu, D. K., Yen, C.-F., Lin, C.-Y., Griffiths, M. D., & Pakpour, A. H. (2021). Expanding Protection Motivation Theory to explain willingness of COVID-19 vaccination uptake among Taiwanese university students. Vaccines, 9, 1046.  Wang, P.-W., Ahorsu, D. K., Lin, C.-Y., Chen, I.-H., Yen, C.-F., Kuo, Y.-J., Griffiths, M. D., & Pakpour, A. H. (2021). Motivation to Have COVID-19 Vaccination Explained Using an Extended Protection Motivation Theory Among University Students in China: The Role of Information Sources. Vaccines, 9, 380. | *Page 25:*  “For example, specific Covid-19 related anxiety and fear of health-related consequences caused by the virus positively predicted the willingness [15, 30, 62].”  *Page 26-27:*  “Recent research applied the Protection Motivation Theory (PMT; [84]) to explain the importance of perceived knowledge about the Covid-19 situation for the vaccination willingness [36, 37]. According to PMT, our self-protection motivation depends on (1) threat appraisal (perceived severity of the threat; perceived personal vulnerability/risk; emotional response to the threat) and (2) coping appraisal (perceived response-efficacy – our belief about the effectiveness of the protective behavior; perceived self-efficacy – our belief about the ability to adopt the protective behavior; perceived costs of the protective behavior) [85]. The most important predictors of the motivation to adapt to the protective behavior are a high level of threat appraisal (specifically severity and vulnerability) and of efficacy (response- and self-), and a low level of perceived costs [86, 87]. Huang, Hung (36) showed that the level of perceived knowledge about Covid-19 vaccines can significantly positively foster the coping appraisal and thus indirectly the motivation for the protective behavior. Wang, Ahorsu (37) emphasized the role of the information source in this context. Specifically, the use of online sources was negatively linked to perceived knowledge, and it was positively associated with greater perceived costs. Other studies that differed between various forms of (online) sources showed that the use of official governmental sites and of traditional media such as television and newspaper reports to get Covid-19 information was positively linked to vaccination willingness [15, 88]. In contrast, the use of social media such as Facebook and Twitter contributed to less willingness [43]. Our results are in line with the available literature. Especially the use of television reports – that are available to the majority of the population in many countries and do not require specific technical or reading skills – seemed to positively contribute to the vaccination willingness in late spring 2021 (positive effect in FR, GE, RU, ES, SV, and UK). The use of newspaper reports was positively linked to willingness in Poland, and the use of official sites provided a positive effect in China and Germany. The use of social media negatively predicted vaccination willingness in Poland, Sweden, and the U.S. The main characteristics of social media are the permanent availability of unfiltered information, the freedom to create and to share online content and to engage in social interaction about this content [89]. However, these characteristics increase the risk of an uncontrollable online sharing of fake news and conspiracy believes about Covid-19 that can negatively impact the knowledge about the vaccines, increase the perceived response cost and thus reduce the motivation to adherence to governmental measures in general [90] and vaccination willingness in particular [91].”  *Page 29:*  “Also, people who have a lower social status and those who life in a large city should be focused on by the companies. It should be emphasized that the vaccination of individuals who do not belong to the Covid-19 risk group and who are not affected by the Covid-19 situation in term of physical health or mental health is also of great importance. They might underestimate the usefulness of mitigation measures including vaccination. If they also underestimate their own risk of infection, they might adhere to the measures to a lesser extent. This conclusion is in line with available literature (e.g., [29, 30]) that applied the Theory of Planned Behavior (TPB; [94]) to explain the Covid-19 vaccination willingness. According to the TPB, attitude, subjective norm, and perceived behavioral control significantly predict our behavioral intention that influences the actual behavior [94]. Recent research from China [29] and Iran [30] showed that in the Covid-19 situation especially perceived risk of infection can foster the attitude toward the vaccination willingness positively. In Taiwan, individuals who felt relatively safe tended to decline the Covid-19 vaccination [31].”  *Page 32:*  “As shown by previous research, the formulation of the question about the willingness can significantly influence the response and it can result in an underestimation of the rate of the vaccination willingness [14].” | Thank you very much for the suggestion of the references. We included the theories that were investigated by the suggested studies in the Discussion section of the revised manuscript. And we discussed the possibilities regarding how to incorporate our findings to these theories to maximize the possibilities in increasing people’s willingness to update Covid-19 vaccine. Moreover, we included the suggested references at further places of the Discussion section. |
| Second, please mention that there is an existing instrument that has been validated and could be use to assess willingness of COVID-19 vaccination uptake.  References:  Yeh, Y.-C., Chen, I.-H., Ahorsu, D. K., Ko, N.-Y., Chen, K.-L., Li, P.-c., Yen, C.-F., Lin, C.-Y., Griffiths, M. D., Pakpour, A. H. (2021). Measurement invariance of the Drivers of COVID-19 Vaccination Acceptance Scale: Comparison between Taiwanese and mainland Chinese-speaking populations. Vaccines, 9(3), 297.  Chen, I.-H., Ahorsu, D. K., Ko, N.-Y., Yen, C.-F., Lin, C.-Y., Griffiths, M. D., Pakpour, A. H. (2021). Adapting the Motors of Influenza Vaccination Acceptance Scale into the Motors of COVID-19 Vaccination Acceptance Scale: Psychometric evaluation among mainland Chinese university students. Vaccine, 39(32), 4510-4515. | *Page 32-33:*  “Sixth, we assessed Covid-19 vaccination willingness with only one item that we formulated for the present study. As shown by previous research, the formulation of the question about the willingness can significantly influence the response and it can result in an underestimation of the rate of the vaccination willingness [14]. Furthermore, even though single-item scales are time and cost efficient instruments [96, 97], future studies should replicate our findings using longer multidimensional measures for the assessment of vaccination willingness. For example, they could use the new developed and validated Drivers of Covid-19 Vaccination Acceptance Scale (DrVac-COVID19S) that consists of four main factors (values, impacts, knowledge, and autonomy) and twelve items [98, 99]. A replication of the current finding with this instrument would contribute to the validation of our single-item measure.” | Thank you very much for the suggestion of both references. Unfortunately, we did not know about this instrument previously. We mentioned that there is an existing validated instrument on the assessment of willingness of Covid-19 vaccination uptake and we included both references in the Limitation section of the revised manuscript. |
| 1. Please ensure that your manuscript meets PLOS ONE's style requirements, including those for file naming. The PLOS ONE style templates can be found at... |  | Our manuscript meets PLOS ONE’s style requirements, including those for file naming. |
| 2. Please provide additional details regarding participant consent. In the Methods section, please ensure that you have specified (1) whether consent was informed and (2) what type you obtained (for instance, written or verbal). If your study included minors, state whether you obtained consent from parents or guardians. If the need for consent was waived by the ethics committee, please include this information. | *Page 8:*  “All participants were properly instructed and gave online their informed consent to participate via an online form.” | In all samples, consent was informed. All data were assessed by an online survey. Therefore, the informed consent was obtained via an online form. This information is included in the Method section of the revised manuscript. No minors were included in the study. |
| 3. Thank you for stating the following in the Acknowledgments Section of your manuscript:  “We acknowledge support by the Open Access Publication Funds of the Ruhr-Universität Bo chum to Julia Brailovskaia (JB). The funders had no role in study design, data collection and analysis, decision to publish, or preparation of the manuscript.”  We note that you have provided funding information within the Acknowledgements Section. Please note that funding information should not appear in the Acknowledgments section or other areas of your manuscript. We will only publish funding information present in the Funding Statement section of the online submission form.  Please remove any funding-related text from the manuscript and let us know how you would like to update your Funding Statement. Currently, your Funding Statement reads as follows:   “We acknowledge support by the Open Access Publication Funds of the Ruhr-Universität Bochum to Julia Brailovskaia (JB) to pay the publication fees. The funders had no role in study design, data collection and analysis, decision to publish, or preparation of the manuscript.”  Please include your amended statements within your cover letter; we will change the online submission form on your behalf. |  | We removed the Acknowledgement section in the revised manuscript. Please include the following Funding Statement:  “We acknowledge support by the Open Access Publication Funds of the Ruhr-Universität Bochum to Julia Brailovskaia (JB). The funders had no role in study design, data collection and analysis, decision to publish, or preparation of the manuscript.” We included this statement within the cover letter. |

| Comment of the Editor/Reviewer | Change – page references refer to the revised manuscript | Our Comment |
| --- | --- | --- |
| *Reviewer 1* |  |  |
| Summary The study aimed to investigate the vaccination willingness and its potential predictors in representative online samples in nine countries (China, France, Germany, Poland, Russia, Spain, Sweden, U.K., U.S.). The overall willingness among the general population was 79%. The highest willingness was in the U.K., followed by Spain and China, the lowest in Russia. Further, factors like demographic variables, mental and physical health status, evaluation of governmental communication, social media use, and general adherence to Covid-19 measures showed a country-specific predictive pattern. However, there still were some notable deficiencies in the article. |  | Thank you very much for the insightful comments. We have considerably revised the original manuscript by addressing each point raised by you, which improved the quality of our manuscript considerably. We really hope to have covered all your suggestions adequately. |
| Introduction  1. Page no.3, Line no. 68: It is better to use general population willingness instead of people’s willingness. | *Page 5:*  “To close the described research gap, we had two main aims: 1.) To investigate the general population willingness for Covid-19 vaccination in the nine countries simultaneously and to compare it between the countries; 2.) To identify factors that could influence the vaccination willingness in the end of spring 2021 – after more than one year of living with the Covid-19 situation and its consequences for everyday life [25, 26].” | We corrected this formulation in the Introduction section of the revised manuscript. |
| 2. The structure of the introduction section is very confusing; the author has first mentioned aim, then kept explaining the literature gap, and then again started talking about aim two. I would suggest restructuring the introduction for better understanding. | *Page 3-7:*  Introduction section | Considering your concern and the concern of the editor, we considerably overworked the structure of the Introduction section in the revised manuscript. Now, we first describe the importance of the Covid-19 vaccination willingness and the literature gap; then, we present the two main aims of our study – 1. To assess the willingness in the nine countries simultaneously and to compare it between the countries, 2. To investigate potential predictors of the willingness; then, we explain the potential predictors that we included in our investigation. We hope that the Introduction is clearer now. |
| Methods and results 3. It is not clear how the author has categorized social status? what is working class? How is it different from other classes in terms of status? | *Page 9:*  “Following previous research on population samples (e.g., [34, 46]), participants were asked…” | Considering the categorization of social status, we followed the conventions of available literature (see e.g., Margraf et al., 2020; Soria et al., 2013; Velten et al., 2021). We included the references in the Method section of the revised manuscript. Please note that for the present investigation the single steps of the classification are not of significant relevance for the results. Therefore, a detailed explanation of the categorization goes beyond the scope of the present study. We hope that you understand this issue.  References:  Margraf, J., Brailovskaia, J., & Schneider, S. (2020). Behavioral measures to fight COVID-19: An 8-country study of perceived usefulness, adherence and their predictors. *PLoS One, 15*(12), e0243523. doi:10.1371/journal.pone.0243523  Soria, K. M., Stebleton, M. J., & Huesman Jr, R. L. (2013). Class counts: Exploring differences in academic and social integration between working-class and middle/upper-class students at large, public research universities. *Journal of College Student Retention: Research, Theory & Practice, 15*(2), 215-242. doi:10.2190/CS.15.2.e  Velten, J., Scholten, S., Brailovskaia, J., & Margraf, J. (2021). Psychometric properties of the S-Scale: Assessing a psychological mindset that mediates the relationship between socioeconomic status and depression. *PLoS One, 16*(10), e0258333. doi:10.1371/journal.pone.0258333 |
| 4. How have the authors validated the COVID-specific questionnaire? | *Page 10:*  “To assess Covid-19 vaccination willingness, participants were asked to answer the question “Have you already been vaccinated against Covid-19 at least once?” using the three options: 1=*No, and I do not want to be vaccinated*, 2=*No, but I would like to be vaccinated*, 3=*Yes*. This question was formulated for the present study. We discussed its wording with experts in health research and medicine.”  *Page 10:*  “Furthermore, following Margraf, Brailovskaia (30) and Brailovskaia, Cosci (40), participants were asked to rate…”  *Page 32-33:*  “Sixth, we assessed Covid-19 vaccination willingness with only one item that we formulated for the present study. As shown by previous research, the formulation of the question about the willingness can significantly influence the response and it can result in an underestimation of the rate of the vaccination willingness [14]. Furthermore, even though single-item scales are time and cost efficient instruments [96, 97], future studies should replicate our findings using longer multidimensional measures for the assessment of vaccination willingness. For example, they could use the new developed and validated Drivers of Covid-19 Vaccination Acceptance Scale (DrVac-COVID19S) that consists of four main factors (values, impacts, knowledge, and autonomy) and twelve items [98, 99]. A replication of the current finding with this instrument would contribute to the validation of our single-item measure.” | We formulated the question about the Covid-19 vaccination willingness for the present study and discussed it with experts in health research and medicine. We included this information in the Method section of the revised manuscript. However, there are no previous studies that validated this question. We acknowledged this point in the Limitation section. The other questions considering Covid-19 were validated by previous research. This information is included in the Method section. |
| 5. What is the response rate in the study? | *Page 8:*  “The response rate was: 92.5% in China, 91.9% in France, 94.5% in Germany, 90.3% in Poland, 92.3% in Russia, 88.7% in Spain, 90.5% in Sweden, 91.1% in the U.K., and 86.4% in the U.S.” | We included the country-specific response rates in the Method section of the revised manuscript. |
| 6. What were all languages used during questionnaire administration? The questionnaire used in the study was in English, or was it translated? | *Page 8:*  “An independent social marketing and research institute (YouGov, [www.yougov.de](http://www.yougov.de)) collected the data via population-based online-panel surveys in the national language of the countries within eight days (May 12 to May 19, 2021).”  *Page 11-12:*  “Previously validated national language versions of the included instruments were used (e.g., PMH: [34, 51]; DASS-21: [52]). In case that no previously validated national language version was available, the international team of the BOOM-Project translated the scales into the national language from the English language version by the customary translation-back-translation-modification procedure [53].” | The online-panel surveys were administrated in the national language of the countries. When available, previously validated national language versions of the used scales were taken (e.g., PMH-Scale: Cai et al., 2017; Margraf et al., 2020; DASS-21: Scholten et al., 2017). In case that no previously validated national language version was available, the measures were translated into the national language from the English language version by the customary translation-back-translation-modification procedure (Berry, 1989) by the international team of our project. We included this information in the method section of the revised manuscript.  References:  Cai, D., Zhu, M., Lin, M., Zhang, X. C., & Margraf, J. (2017). The bidirectional relationship between positive mental health and social rhythm in college students: a three-year longitudinal study. *Frontiers in Psychology, 8*, 1-7. doi:10.3389/fpsyg.2017.01119  Margraf, J., Brailovskaia, J., & Schneider, S. (2020). Behavioral measures to fight COVID-19: An 8-country study of perceived usefulness, adherence and their predictors. *PLoS One, 15*(12), e0243523. doi:10.1371/journal.pone.0243523  Scholten, S., Velten, J., Bieda, A., Zhang, X. C., & Margraf, J. (2017). Testing measurement invariance of the Depression, Anxiety, and Stress Scales (DASS-21) across four countries. *Psychological Assessment, 29*(11), 1376-1390. doi:10.1037/pas0000440 |
| 7. Please specify n.s in table number 3 | *Page 16:*  Table 3, “Notes: Cramer’s V is presented for significant comparisons; n.s.=not significant comparison” | We apologize that we did not specify the abbreviation n.s. It stands for “not significant” comparison. Note that the effect size Cramer’s V was calculated only for significant comparisons.  We specified n.s. and included this explanation in the revised manuscript. |
| Discussion  8. What will be the possible reason for the lower willingness level among China in this study? Line 389-391, page no.19 | *Page 20-21:*  “Only in China, we found a slightly lower willingness level (difference: about 1.7%) that however was higher than the figures reported by Chen, Li (10) and Liu, Zhang (11) in 2020.” | Please note that the difference between the willingness level in our study and the one described by Lazarus et al. (2020) in China was very small (88.6% - 86.9% = 1.7%). Following recent research from Taiwan (Kukreti et al., 2021), it might be that due to the rather low infection rates in China the participants from this country felt less at risk for Covid-19 and therefore revealed a lower vaccination willingness in 2021. However, considering that the difference between earlier and our findings is so low, we prefer not to emphasize this finding and not to speculate about potential reasons for the difference. We think that it is important to mention the difference, but also to show that it is very low. We included the difference in the revised manuscript.  References:  Kukreti, S., Lu, M.-Y., Lin, Y.-H., Strong, C., Lin, C.-Y., Ko, N.-Y., . . . Ko, W.-C. (2021). Willingness of Taiwan’s healthcare workers and outpatients to vaccinate against COVID-19 during a period without community outbreaks. *Vaccines, 9*(3), 246-255. doi:10.3390/vaccines9030246  Lazarus, J. V., Wyka, K., Rauh, L., Rabin, K., Ratzan, S., Gostin, L. O., . . . El-Mohandes, A. (2020). Hesitant or Not? The Association of Age, Gender, and Education with Potential Acceptance of a COVID-19 Vaccine: A Country-level Analysis. *Journal of Health Communication, 25*(10), 799-807. doi:10.1080/10810730.2020.1868630 |
| 9. What does the author mean by physical intervention here? Line411, page no.20 | *Page 21:*  “Thus, it seems that measures that do not refer to invasive interventions are evaluated differently in France than these that include invasive steps such as vaccination. Invasive interventions might be linked to more safety concerns than for example the wearing of face masks or the keeping of distance [57], and thus could meet a higher level of rejection in France.” | We apologize the unclear formulation. We mean invasive interventions or steps such as the vaccination. We reformulated this section in the Discussion section of the revised manuscript. |
| 10. What does the author mean by correlative design here? | *Page 32:*  “First, all data were assessed at the same measurement time point. The cross-sectional design of the present study does not allow true conclusions on causality. Therefore, the effectiveness of our country-specific suggestions for the improvement of the vaccination willingness should be investigated by future experimental research.” | We apologize the unclear formulation. We assessed all data at the same measurement time point. This design is termed as correlative or cross-sectional (see Kraemer et al., 1997). We explained this and changed the term correlative to cross-sectional in the revised manuscript.  Reference:  Kraemer, H. C., Kazdin, A. E., Offord, D. R., Kessler, R. C., Jensen, P. S., & Kupfer, D. J. (1997). Coming to terms with the terms of risk. *Archives of General Psychiatry, 54*(4), 337-343. doi:10.1001/archpsyc.1997.01830160065009 |
