# Supplementary material for: To vaccinate or not to vaccinate!? Predictors of willingness to receive Covid-19 vaccination in Europe, the U.S., and China
Source: PLoS One. 2021 Dec 1;16(12):e0260230. doi: 10.1371/journal.pone.0260230 (PMC8635370; doi:10.1371/journal.pone.0260230)
Supplement: Peer review history

# Peer review: Decision Letter 0

**DOI:** 10.1371/journal.pone.0260230.r001

**Authors:** Chung-Ying Lin

1 Nov 2021

PONE-D-21-31209To vaccinate or not to vaccinate!? Predictors of willingness to receive Covid-19 vaccination in Europe, the U.S., and ChinaPLOS ONE

Dear Dr. Brailovskaia,

Thank you for submitting your manuscript to PLOS ONE. After careful consideration, we feel that it has merit but does not fully meet PLOS ONE’s publication criteria as it currently stands. Therefore, we invite you to submit a revised version of the manuscript that addresses the points raised during the review process.

==============================

The cross-country comparisons of the vaccination uptake willingness is important as we know that the herd immunity should be better to achieve around the world. Then, we can have more country interaction and reactivate the economic communications. Therefore, I believe that the present study tackle an important and timely topic. An expert in this area has reviewed this work and provides good comments for you to work on. In general, I agree with all of her opinions and hope that all these comments can help you improve the presentation. I would especially follow her suggestion regarding the Introduction structure. Indeed, please put all the aims, purposes, and hypotheses toward the end of the Introduction. In this way, the readability can be improved. Moreover, I would like the authors to mention the following issues in the Introduction. First, please mention that there is some debate regarding the willingness of COVID-19 vaccination uptake with relevant ciations (e.g., Kukreti et al. found some low levels of willingness in some populations, while Rieger argues that willingness of COVID-19 vaccination uptake might be underestimated). Second, please mention that there may have inequity issue for the vaccination. References:Kukreti, S., Lu, M.-Y., Lin, Y.-H., Strong, C., Lin, C.-Y., Ko, N.-Y., Chen, P.-L., & Ko, W.-C. (2021). Willingness of Taiwan’s healthcare workers and outpatients to vaccinate against COVID-19 during a period without community outbreaks. Vaccines, 9(3), 246. Rieger MO. Willingness to vaccinate against COVID-19 might be systematically underestimated. Asian J Soc Health Behav 2021;4:81-3.Alimoradi Z, Lin CY, Pakpour AH. Coronavirus disease-19 vaccine inequity and gross domestic product. Asian J Soc Health Behav 2021;4:129-30Apart from the Introduction, please mention in the Discussion regarding the following two points.First, there are existing behavior theories that have been tested to understand the vaccination uptake. The authors should discuss the possibilities regarding how to incorporate their predictor findings to these theories to maximize the possibilities in increasing people's willingness to uptake COVID-19 vaccine. Please see the following references:Fan, C.-W., Chen, I.-H., Ko, N.-Y., Yen, C.-F., Lin, C.-Y., Griffiths, M. D., & Pakpour, A. H. (2021).  Extended theory of planned behavior in explaining the intention to COVID-19 vaccination uptake among mainland Chinese university students: An online survey study. Human Vaccines & Immunotherapeutics, 17(10), 3413-3420.Yahaghi, R., Ahmadizade, S., Fotuhi, R., Taherkhani, E., Ranjbaran, M., Buchali, Z., Jafari, R., Zamani, N., Shahbazkhania, A., Simiari, H., Rahmani, J., Yazdi, N., Alijani, H., Poorzolfaghar, L., Rajabi, F., Lin, C.-Y., Broström, A., Griffiths, M. D., Pakpour, A. H. (2021). Fear of COVID-19 and perceived COVID-19 infectability supplement Theory of Planned Behavior to explain Iranians’ intention to get COVID-19 vaccinated. Vaccines, 9, 684. Huang, P.-C., Hung, C.-H., Kuo, Y.-J., Chen, Y.-P., Ahorsu, D. K., Yen, C.-F., Lin, C.-Y., Griffiths, M. D., & Pakpour, A. H. (2021). Expanding Protection Motivation Theory to explain willingness of COVID-19 vaccination uptake among Taiwanese university students. Vaccines, 9, 1046. Wang, P.-W., Ahorsu, D. K., Lin, C.-Y., Chen, I.-H., Yen, C.-F., Kuo, Y.-J., Griffiths, M. D., & Pakpour, A. H. (2021). Motivation to Have COVID-19 Vaccination Explained Using an Extended Protection Motivation Theory Among University Students in China: The Role of Information Sources. Vaccines, 9, 380.Second, please mention that there is an existing instrument that has been validated and could be use to assess willingness of COVID-19 vaccination uptake.References:Yeh, Y.-C., Chen, I.-H., Ahorsu, D. K., Ko, N.-Y., Chen, K.-L., Li, P.-c., Yen, C.-F., Lin, C.-Y., Griffiths, M. D., Pakpour, A. H. (2021). Measurement invariance of the Drivers of COVID-19 Vaccination Acceptance Scale: Comparison between Taiwanese and mainland Chinese-speaking populations. Vaccines, 9(3), 297. Chen, I.-H., Ahorsu, D. K., Ko, N.-Y., Yen, C.-F., Lin, C.-Y., Griffiths, M. D., Pakpour, A. H. (2021). Adapting the Motors of Influenza Vaccination Acceptance Scale into the Motors of COVID-19 Vaccination Acceptance Scale: Psychometric evaluation among mainland Chinese university students. Vaccine, 39(32), 4510-4515.

==============================

Please submit your revised manuscript by Dec 16 2021 11:59PM. If you will need more time than this to complete your revisions, please reply to this message or contact the journal office at plosone@plos.org. When you're ready to submit your revision, log on to https://www.editorialmanager.com/pone/ and select the 'Submissions Needing Revision' folder to locate your manuscript file.

Please include the following items when submitting your revised manuscript:

- A rebuttal letter that responds to each point raised by the academic editor and reviewer(s). You should upload this letter as a separate file labeled 'Response to Reviewers'.
- A marked-up copy of your manuscript that highlights changes made to the original version. You should upload this as a separate file labeled 'Revised Manuscript with Track Changes'.
- An unmarked version of your revised paper without tracked changes. You should upload this as a separate file labeled 'Manuscript'.

If you would like to make changes to your financial disclosure, please include your updated statement in your cover letter. Guidelines for resubmitting your figure files are available below the reviewer comments at the end of this letter.

If applicable, we recommend that you deposit your laboratory protocols in protocols.io to enhance the reproducibility of your results. Protocols.io assigns your protocol its own identifier (DOI) so that it can be cited independently in the future. For instructions see: https://journals.plos.org/plosone/s/submission-guidelines#loc-laboratory-protocols. Additionally, PLOS ONE offers an option for publishing peer-reviewed Lab Protocol articles, which describe protocols hosted on protocols.io. Read more information on sharing protocols at https://plos.org/protocols?utm_medium=editorial-email&utm_source=authorletters&utm_campaign=protocols.

We look forward to receiving your revised manuscript.

Kind regards,

Chung-Ying Lin

Academic Editor

PLOS ONE

Journal Requirements:

When submitting your revision, we need you to address these additional requirements.

1. Please ensure that your manuscript meets PLOS ONE's style requirements, including those for file naming. The PLOS ONE style templates can be found at

https://journals.plos.org/plosone/s/file?id=wjVg/PLOSOne_formatting_sample_main_body.pdf and

https://journals.plos.org/plosone/s/file?id=ba62/PLOSOne_formatting_sample_title_authors_affiliations.pdf

2. Please provide additional details regarding participant consent. In the Methods section, please ensure that you have specified (1) whether consent was informed and (2) what type you obtained (for instance, written or verbal). If your study included minors, state whether you obtained consent from parents or guardians. If the need for consent was waived by the ethics committee, please include this information.

3. Thank you for stating the following in the Acknowledgments Section of your manuscript:

“We acknowledge support by the Open Access Publication Funds of the Ruhr-Universität Bo chum to Julia Brailovskaia (JB). The funders had no role in study design, data collection and analysis, decision to publish, or preparation of the manuscript.”

We note that you have provided funding information within the Acknowledgements Section. Please note that funding information should not appear in the Acknowledgments section or other areas of your manuscript. We will only publish funding information present in the Funding Statement section of the online submission form.

Please remove any funding-related text from the manuscript and let us know how you would like to update your Funding Statement. Currently, your Funding Statement reads as follows:

 “We acknowledge support by the Open Access Publication Funds of the Ruhr-Universität Bochum to Julia Brailovskaia (JB) to pay the publication fees. The funders had no role in study design, data collection and analysis, decision to publish, or preparation of the manuscript.”

Please include your amended statements within your cover letter; we will change the online submission form on your behalf.

[Note: HTML markup is below. Please do not edit.]

Reviewers' comments:

Reviewer's Responses to Questions

**Comments to the Author**

1. Is the manuscript technically sound, and do the data support the conclusions?

The manuscript must describe a technically sound piece of scientific research with data that supports the conclusions. Experiments must have been conducted rigorously, with appropriate controls, replication, and sample sizes. The conclusions must be drawn appropriately based on the data presented. 

Reviewer #1: Yes

**********

2. Has the statistical analysis been performed appropriately and rigorously? 

Reviewer #1: Yes

**********

3. Have the authors made all data underlying the findings in their manuscript fully available?

The PLOS Data policy requires authors to make all data underlying the findings described in their manuscript fully available without restriction, with rare exception (please refer to the Data Availability Statement in the manuscript PDF file). The data should be provided as part of the manuscript or its supporting information, or deposited to a public repository. For example, in addition to summary statistics, the data points behind means, medians and variance measures should be available. If there are restrictions on publicly sharing data—e.g. participant privacy or use of data from a third party—those must be specified.

Reviewer #1: Yes

**********

4. Is the manuscript presented in an intelligible fashion and written in standard English?

PLOS ONE does not copyedit accepted manuscripts, so the language in submitted articles must be clear, correct, and unambiguous. Any typographical or grammatical errors should be corrected at revision, so please note any specific errors here.

Reviewer #1: No

**********

5. Review Comments to the Author

Please use the space provided to explain your answers to the questions above. You may also include additional comments for the author, including concerns about dual publication, research ethics, or publication ethics. (Please upload your review as an attachment if it exceeds 20,000 characters)

Reviewer #1: Comments to the Author

Summary

The study aimed to investigate the vaccination willingness and its potential predictors in representative online samples in nine countries (China, France, Germany, Poland, Russia, Spain, Sweden, U.K., U.S.). The overall willingness among the general population was 79%. The highest willingness was in the U.K., followed by Spain and China, the lowest in Russia. Further, factors like demographic variables, mental and physical health status, evaluation of governmental communication, social media use, and general adherence to Covid-19 measures showed a country-specific predictive pattern.

However, there still were some notable deficiencies in the article.

Introduction

1. Page no.3, Line no. 68: It is better to use general population willingness instead of people’s willingness.

2. The structure of the introduction section is very confusing; the author has first mentioned aim, then kept explaining the literature gap, and then again started talking about aim two. I would suggest restructuring the introduction for better understanding.

Methods and results

3. It is not clear how the author has categorized social status? what is working class? How is it different from other classes in terms of status?

4. How have the authors validated the COVID-specific questionnaire?

5. What is the response rate in the study?

6. What were all languages used during questionnaire administration? The questionnaire used in the study was in English, or was it translated?

7. Please specify n.s in table number 3

Discussion

8. What will be the possible reason for the lower willingness level among China in this study? Line 389-391, page no.19

9. What does the author mean by physical intervention here? Line411, page no.20

10. What does the author mean by correlative design here?

**********

6. PLOS authors have the option to publish the peer review history of their article (what does this mean?). If published, this will include your full peer review and any attached files.

If you choose “no”, your identity will remain anonymous but your review may still be made public.

**Do you want your identity to be public for this peer review?** For information about this choice, including consent withdrawal, please see our Privacy Policy.

Reviewer #1: No

[NOTE: If reviewer comments were submitted as an attachment file, they will be attached to this email and accessible via the submission site. Please log into your account, locate the manuscript record, and check for the action link "View Attachments". If this link does not appear, there are no attachment files.]

While revising your submission, please upload your figure files to the Preflight Analysis and Conversion Engine (PACE) digital diagnostic tool, https://pacev2.apexcovantage.com/. PACE helps ensure that figures meet PLOS requirements. To use PACE, you must first register as a user. Registration is free. Then, login and navigate to the UPLOAD tab, where you will find detailed instructions on how to use the tool. If you encounter any issues or have any questions when using PACE, please email PLOS at figures@plos.org. Please note that Supporting Information files do not need this step.

Attachment Submitted filename: Review_PONE-D-21-31209.docx Click here for additional data file.

# Author comment: Author response to Decision Letter 0

**DOI:** 10.1371/journal.pone.0260230.r002

3 Nov 2021

Editor:

The cross-country comparisons of the vaccination uptake willingness is important as we know that the herd immunity should be better to achieve around the world. Then, we can have more country interaction and reactivate the economic communications. Therefore, I believe that the present study tackle an important and timely topic. An expert in this area has reviewed this work and provides good comments for you to work on. In general, I agree with all of her opinions and hope that all these comments can help you improve the presentation. 

Response to Editor:

Thank you very much for the possibility to submit a revised version of our manuscript. We have considerably revised the original manuscript by addressing each point raised by you and the reviewer, which improved the quality of our manuscript considerably. We really hope to have covered all the suggestions adequately.

1. I would especially follow her suggestion regarding the Introduction structure. Indeed, please put all the aims, purposes, and hypotheses toward the end of the Introduction. In this way, the readability can be improved. 

Response to 1.:

Considering your concern and the concern of the reviewer, we considerably overworked the structure of the Introduction section in the revised manuscript. Thank you very much for the suggested references. We have incorporated the different references in the overworked Introduction section. They strengthened our work. Now, we first describe the importance of the Covid-19 vaccination willingness and the literature gap; then, we present the two main aims of our study – 1. To assess the willingness in the nine countries simultaneously and to compare it between the countries, 2. To investigate potential predictors of the willingness; then, we explain the potential predictors that we included in our investigation. We hope that the Introduction section is clearer now.

2. Moreover, I would like the authors to mention the following issues in the Introduction. First, please mention that there is some debate regarding the willingness of COVID-19 vaccination uptake with relevant ciations (e.g., Kukreti et al. found some low levels of willingness in some populations, while Rieger argues that willingness of COVID-19 vaccination uptake might be underestimated). Second, please mention that there may have inequity issue for the vaccination. 

References:

Kukreti, S., Lu, M.-Y., Lin, Y.-H., Strong, C., Lin, C.-Y., Ko, N.-Y., Chen, P.-L., & Ko, W.-C. (2021). Willingness of Taiwan’s healthcare workers and outpatients to vaccinate against COVID-19 during a period without community outbreaks. Vaccines, 9(3), 246. 

Rieger MO. Willingness to vaccinate against COVID-19 might be systematically underestimated. Asian J Soc Health Behav 2021;4:81-3.

Alimoradi Z, Lin CY, Pakpour AH. Coronavirus disease-19 vaccine inequity and gross domestic product. Asian J Soc Health Behav 2021;4:129-30

Response to 2.:

Following your advice and the advice of the reviewer, we considerably overworked the structure of the Introduction section in the revised manuscript. Thank you very much for the suggestion of the three references. We included all of them in the overworked Introduction section. Moreover, we included them also in the Discussion section. 

3. Apart from the Introduction, please mention in the Discussion regarding the following two points.

First, there are existing behavior theories that have been tested to understand the vaccination uptake. The authors should discuss the possibilities regarding how to incorporate their predictor findings to these theories to maximize the possibilities in increasing people's willingness to uptake COVID-19 vaccine. Please see the following references:

Fan, C.-W., Chen, I.-H., Ko, N.-Y., Yen, C.-F., Lin, C.-Y., Griffiths, M. D., & Pakpour, A. H. (2021). Extended theory of planned behavior in explaining the intention to COVID-19 vaccination uptake among mainland Chinese university students: An online survey study. Human Vaccines & Immunotherapeutics, 17(10), 3413-3420.

Yahaghi, R., Ahmadizade, S., Fotuhi, R., Taherkhani, E., Ranjbaran, M., Buchali, Z., Jafari, R., Zamani, N., Shahbazkhania, A., Simiari, H., Rahmani, J., Yazdi, N., Alijani, H., Poorzolfaghar, L., Rajabi, F., Lin, C.-Y., Broström, A., Griffiths, M. D., Pakpour, A. H. (2021). Fear of COVID-19 and perceived COVID-19 infectability supplement Theory of Planned Behavior to explain Iranians’ intention to get COVID-19 vaccinated. Vaccines, 9, 684. 

Huang, P.-C., Hung, C.-H., Kuo, Y.-J., Chen, Y.-P., Ahorsu, D. K., Yen, C.-F., Lin, C.-Y., Griffiths, M. D., & Pakpour, A. H. (2021). Expanding Protection Motivation Theory to explain willingness of COVID-19 vaccination uptake among Taiwanese university students. Vaccines, 9, 1046. 

Wang, P.-W., Ahorsu, D. K., Lin, C.-Y., Chen, I.-H., Yen, C.-F., Kuo, Y.-J., Griffiths, M. D., & Pakpour, A. H. (2021). Motivation to Have COVID-19 Vaccination Explained Using an Extended Protection Motivation Theory Among University Students in China: The Role of Information Sources. Vaccines, 9, 380.

Response to 3.:

Thank you very much for the suggestion of the references. We included the theories that were investigated by the suggested studies in the Discussion section of the revised manuscript. And we discussed the possibilities regarding how to incorporate our findings to these theories to maximite the possibilities in increasing people’s willingness to update Covid-19 vaccine. Moreover, we included the suggested references at further places of the Discussion section.

4. Second, please mention that there is an existing instrument that has been validated and could be use to assess willingness of COVID-19 vaccination uptake.

References:

Yeh, Y.-C., Chen, I.-H., Ahorsu, D. K., Ko, N.-Y., Chen, K.-L., Li, P.-c., Yen, C.-F., Lin, C.-Y., Griffiths, M. D., Pakpour, A. H. (2021). Measurement invariance of the Drivers of COVID-19 Vaccination Acceptance Scale: Comparison between Taiwanese and mainland Chinese-speaking populations. Vaccines, 9(3), 297. 

Chen, I.-H., Ahorsu, D. K., Ko, N.-Y., Yen, C.-F., Lin, C.-Y., Griffiths, M. D., Pakpour, A. H. (2021). Adapting the Motors of Influenza Vaccination Acceptance Scale into the Motors of COVID-19 Vaccination Acceptance Scale: Psychometric evaluation among mainland Chinese university students. Vaccine, 39(32), 4510-4515.

Response to 4.:

Thank you very much for the suggestion of both references. Unfortunately, we did not know about this instrument previously. We mentioned that there is an existing validated instrument on the assessment of willingness of Covid-19 vaccination uptake and we included both references in the Limitation section of the revised manuscript.

5. 1. Please ensure that your manuscript meets PLOS ONE's style requirements, including those for file naming. The PLOS ONE style templates can be found at...

Response to 5.:

Our manuscript meets PLOS ONE’s style requirements, including those for file naming.

6. 2. Please provide additional details regarding participant consent. In the Methods section, please ensure that you have specified (1) whether consent was informed and (2) what type you obtained (for instance, written or verbal). If your study included minors, state whether you obtained consent from parents or guardians. If the need for consent was waived by the ethics committee, please include this information.

Response to 6.:

In all samples, consent was informed. All data were assessed by an online survey. Therefore, the informed consent was obtained via an online form. This information is included in the Method section of the revised manuscript. No minors were included in the study.

7. 3. Thank you for stating the following in the Acknowledgments Section of your manuscript:

“We acknowledge support by the Open Access Publication Funds of the Ruhr-Universität Bo chum to Julia Brailovskaia (JB). The funders had no role in study design, data collection and analysis, decision to publish, or preparation of the manuscript.”

We note that you have provided funding information within the Acknowledgements Section. Please note that funding information should not appear in the Acknowledgments section or other areas of your manuscript. We will only publish funding information present in the Funding Statement section of the online submission form.

Please remove any funding-related text from the manuscript and let us know how you would like to update your Funding Statement. Currently, your Funding Statement reads as follows:

 “We acknowledge support by the Open Access Publication Funds of the Ruhr-Universität Bochum to Julia Brailovskaia (JB) to pay the publication fees. The funders had no role in study design, data collection and analysis, decision to publish, or preparation of the manuscript.”

Please include your amended statements within your cover letter; we will change the online submission form on your behalf.

Response to 7.:

We removed the Acknowledgement section in the revised manuscript. Please include the following Funding Statement:

“We acknowledge support by the Open Access Publication Funds of the Ruhr-Universität Bochum to Julia Brailovskaia (JB). The funders had no role in study design, data collection and analysis, decision to publish, or preparation of the manuscript.” We included this statement within the cover letter.

Reviewer:

Summary

The study aimed to investigate the vaccination willingness and its potential predictors in representative online samples in nine countries (China, France, Germany, Poland, Russia, Spain, Sweden, U.K., U.S.). The overall willingness among the general population was 79%. The highest willingness was in the U.K., followed by Spain and China, the lowest in Russia. Further, factors like demographic variables, mental and physical health status, evaluation of governmental communication, social media use, and general adherence to Covid-19 measures showed a country-specific predictive pattern.

However, there still were some notable deficiencies in the article.

Response to Reviewer:

Thank you very much for the insightful comments. We have considerably revised the original manuscript by addressing each point raised by you, which improved the quality of our manuscript considerably. We really hope to have covered all your suggestions adequately.

1. Introduction

Page no.3, Line no. 68: It is better to use general population willingness instead of people’s willingness.

Response to 1.:

We corrected this formulation in the Introduction section of the revised manuscript.

2. The structure of the introduction section is very confusing; the author has first mentioned aim, then kept explaining the literature gap, and then again started talking about aim two. I would suggest restructuring the introduction for better understanding.

Response to 2.:

Considering your concern and the concern of the editor, we considerably overworked the structure of the Introduction section in the revised manuscript. Now, we first describe the importance of the Covid-19 vaccination willingness and the literature gap; then, we present the two main aims of our study – 1. To assess the willingness in the nine countries simultaneously and to compare it between the countries, 2. To investigate potential predictors of the willingness; then, we explain the potential predictors that we included in our investigation. We hope that the Introduction is clearer now.

3. Methods and results

It is not clear how the author has categorized social status? what is working class? How is it different from other classes in terms of status?

Response to 3.:

Considering the categorization of social status, we followed the conventions of available literature (see e.g., Margraf et al., 2020; Soria et al., 2013; Velten et al., 2021). We included the references in the Method section of the revised manuscript. Please note that for the present investigation the single steps of the classification are not of significant relevance for the results. Therefore, a detailed explanation of the categorization goes beyond the scope of the present study. We hope that you understand this issue.

References:

Margraf, J., Brailovskaia, J., & Schneider, S. (2020). Behavioral measures to fight COVID-19: An 8-country study of perceived usefulness, adherence and their predictors. PLoS One, 15(12), e0243523. doi:10.1371/journal.pone.0243523

Soria, K. M., Stebleton, M. J., & Huesman Jr, R. L. (2013). Class counts: Exploring differences in academic and social integration between working-class and middle/upper-class students at large, public research universities. Journal of College Student Retention: Research, Theory & Practice, 15(2), 215-242. doi:10.2190/CS.15.2.e

Velten, J., Scholten, S., Brailovskaia, J., & Margraf, J. (2021). Psychometric properties of the S-Scale: Assessing a psychological mindset that mediates the relationship between socioeconomic status and depression. PLoS One, 16(10), e0258333. doi:10.1371/journal.pone.0258333

4. How have the authors validated the COVID-specific questionnaire?

Response to 4.:

We formulated the question about the Covid-19 vaccination willingness for the present study and discussed it with experts in health research and medicine. We included this information in the Method section of the revised manuscript. However, there are no previous studies that validated this question. We acknowledged this point in the Limitation section. The other questions considering Covid-19 were validated by previous research. This information is included in the Method section.

5. What is the response rate in the study?

Response to 5.:

We included the country-specific response rates in the Method section of the revised manuscript.

6. What were all languages used during questionnaire administration? The questionnaire used in the study was in English, or was it translated?

Response to 6.:

The online-panel surveys were administrated in the national language of the countries. When available, previously validated national language versions of the used scales were taken (e.g., PMH-Scale: Cai et al., 2017; Margraf et al., 2020; DASS-21: Scholten et al., 2017). In case that no previously validated national language version was available, the measures were translated into the national language from the English language version by the customary translation-back-translation-modification procedure (Berry, 1989) by the international team of our project. We included this information in the method section of the revised manuscript. 

References:

Cai, D., Zhu, M., Lin, M., Zhang, X. C., & Margraf, J. (2017). The bidirectional relationship between positive mental health and social rhythm in college students: a three-year longitudinal study. Frontiers in Psychology, 8, 1-7. doi:10.3389/fpsyg.2017.01119

Margraf, J., Brailovskaia, J., & Schneider, S. (2020). Behavioral measures to fight COVID-19: An 8-country study of perceived usefulness, adherence and their predictors. PLoS One, 15(12), e0243523. doi:10.1371/journal.pone.0243523

Scholten, S., Velten, J., Bieda, A., Zhang, X. C., & Margraf, J. (2017). Testing measurement invariance of the Depression, Anxiety, and Stress Scales (DASS-21) across four countries. Psychological Assessment, 29(11), 1376-1390. doi:10.1037/pas0000440

7. Please specify n.s in table number 3

Response to 7.:

We apologize that we did not specify the abbreviation n.s. It stands for “not significant” comparison. Note that the effect size Cramer’s V was calculated only for significant comparisons.

We specified n.s. and included this explanation in the revised manuscript. 

8. Discussion

What will be the possible reason for the lower willingness level among China in this study? Line 389-391, page no.19

Response to 8.:

Please note that the difference between the willingness level in our study and the one described by Lazarus et al. (2020) in China was very small (88.6% - 86.9% = 1.7%). Following recent research from Taiwan (Kukreti et al., 2021), it might be that due to the rather low infection rates in China the participants from this country felt less at risk for Covid-19 and therefore revealed a lower vaccination willingness in 2021. However, considering that the difference between earlier and our findings is so low, we prefer not to emphasize this finding and not to speculate about potential reasons for the difference. We think that it is important to mention the difference, but also to show that it is very low. We included the difference in the revised manuscript.

References:

Kukreti, S., Lu, M.-Y., Lin, Y.-H., Strong, C., Lin, C.-Y., Ko, N.-Y., . . . Ko, W.-C. (2021). Willingness of Taiwan’s healthcare workers and outpatients to vaccinate against COVID-19 during a period without community outbreaks. Vaccines, 9(3), 246-255. doi:10.3390/vaccines9030246

Lazarus, J. V., Wyka, K., Rauh, L., Rabin, K., Ratzan, S., Gostin, L. O., . . . El-Mohandes, A. (2020). Hesitant or Not? The Association of Age, Gender, and Education with Potential Acceptance of a COVID-19 Vaccine: A Country-level Analysis. Journal of Health Communication, 25(10), 799-807. doi:10.1080/10810730.2020.1868630

9. What does the author mean by physical intervention here? Line411, page no.20

Response to 9.:

We apologize the unclear formulation. We mean invasive interventions or steps such as the vaccination. We reformulated this section in the Discussion section of the revised manuscript.

10. What does the author mean by correlative design here?

Response to 10.:

We apologize the unclear formulation. We assessed all data at the same measurement time point. This design is termed as correlative or cross-sectional (see Kraemer et al., 1997). We explained this and changed the term correlative to cross-sectional in the revised manuscript.

Reference:

Kraemer, H. C., Kazdin, A. E., Offord, D. R., Kessler, R. C., Jensen, P. S., & Kupfer, D. J. (1997). Coming to terms with the terms of risk. Archives of General Psychiatry, 54(4), 337-343. doi:10.1001/archpsyc.1997.01830160065009

Attachment Submitted filename: Response to Reviewers.docx Click here for additional data file.

# Editor report: Decision Letter 1

**DOI:** 10.1371/journal.pone.0260230.r003

**Authors:** Chung-Ying Lin

5 Nov 2021

To vaccinate or not to vaccinate!? Predictors of willingness to receive Covid-19 vaccination in Europe, the U.S., and China

PONE-D-21-31209R1

Dear Dr. Brailovskaia,

We’re pleased to inform you that your manuscript has been judged scientifically suitable for publication and will be formally accepted for publication once it meets all outstanding technical requirements.

Within one week, you’ll receive an e-mail detailing the required amendments. When these have been addressed, you’ll receive a formal acceptance letter and your manuscript will be scheduled for publication.

An invoice for payment will follow shortly after the formal acceptance. To ensure an efficient process, please log into Editorial Manager at http://www.editorialmanager.com/pone/, click the 'Update My Information' link at the top of the page, and double check that your user information is up-to-date. If you have any billing related questions, please contact our Author Billing department directly at authorbilling@plos.org.

If your institution or institutions have a press office, please notify them about your upcoming paper to help maximize its impact. If they’ll be preparing press materials, please inform our press team as soon as possible -- no later than 48 hours after receiving the formal acceptance. Your manuscript will remain under strict press embargo until 2 pm Eastern Time on the date of publication. For more information, please contact onepress@plos.org.

Kind regards,

Chung-Ying Lin

Academic Editor

PLOS ONE

Additional Editor Comments (optional):

I especially want to thank the authors taking all the comments from the reviewer and I seriously. I can tell that the authors have substantially improve their study via the revised manuscript. I am happy to see the important paper to be published. Congrats!

Reviewers' comments:

# Editor report: Acceptance letter

**DOI:** 10.1371/journal.pone.0260230.r004

**Authors:** Chung-Ying Lin

16 Nov 2021

PONE-D-21-31209R1 

To vaccinate or not to vaccinate!? Predictors of willingness to receive Covid-19 vaccination in Europe, the U.S., and China 

Dear Dr. Brailovskaia:

I'm pleased to inform you that your manuscript has been deemed suitable for publication in PLOS ONE. Congratulations! Your manuscript is now with our production department. 

If your institution or institutions have a press office, please let them know about your upcoming paper now to help maximize its impact. If they'll be preparing press materials, please inform our press team within the next 48 hours. Your manuscript will remain under strict press embargo until 2 pm Eastern Time on the date of publication. For more information please contact onepress@plos.org.

If we can help with anything else, please email us at plosone@plos.org. 

Thank you for submitting your work to PLOS ONE and supporting open access. 

Kind regards, 

PLOS ONE Editorial Office Staff

on behalf of

Dr. Chung-Ying Lin 

Academic Editor

PLOS ONE
